# Chemical Variations among Shengmaisan-Based TCM Patent Drugs by Ultra-High Performance Liquid Chromatography Coupled with Hybrid Quadrupole Orbitrap Mass Spectrometry

**DOI:** 10.3390/molecules26134000

**Published:** 2021-06-30

**Authors:** Lulu Xu, Zhanpeng Shang, Yungang Tian, Ming Xiong, Dilaram Nijat, Yuan Wang, Xue Qiao, Min Ye

**Affiliations:** 1 State Key Laboratory of Natural and Biomimetic Drugs, School of Pharmaceutical Sciences, Peking University, 38 Xueyuan Road, Beijing 100191, China; xll@bucm.edu.cn (L.X.); zpshang1206@bjmu.edu.cn (Z.S.); 20400220@muc.edu.cn (Y.T.); 1810307206@pku.edu.cn (M.X.); dilaram@bjmu.edu.cn (D.N.); wangyuan@bjmu.edu.cn (Y.W.); 2 School of Chinese Materia Medica, Beijing University of Chinese Medicine, Beijing 100029, China; 3 Department of Integration of Chinese and Western Medicine, School of Basic Medical Sciences, Peking University, Beijing 100191, China; 4 Key Laboratory of Molecular Cardiovascular Sciences of Ministry of Education, Peking University, Beijing 100191, China

**Keywords:** Shengmaisan (SMS), TCM patent drugs, chemical variation, UHPLC/Orbitrap-MS

## Abstract

Shengmaisan (SMS) is a famous traditional Chinese medicine (TCM) formula to treat coronary heart diseases. It has been developed into several TCM patent drugs to meet the demands of different patients. In this study, a research strategy was proposed to reveal the chemical variations among four SMS-based patent drugs, including Shengmai Oral Solution (Shengmaiyin, SMY), Shengmai Capsule (Shengmai Jiaonang, SMJN), Yiqi Fumai Injection (YQFMI), and Yiqi Fumai Capsule (Yiqi Fumai Jiaonang, YQJN). Firstly, 227 compounds were tentatively identified using an Orbitrap-MS in the full scan/dd-MS^2^ mode. Secondly, untargeted metabolomics analysis suggested that ginsenosides, steroidal saponins, and lignans were the main types of differential compounds for the four patent drugs. Finally, the contents of 25 compounds were simultaneously determined in 30 batches of samples in the parallel reaction monitoring (PRM) mode. Partial least squares discriminant analysis (PLS-DA) revealed the contents of ginsenosides Re, Rg1, Rb1, Ro, and Rg3, and schisandrin showed the highest intergroup variations. These compounds were chemical markers to differentiate the SMS-based patent drugs.

## 1. Introduction

Traditional Chinese medicines (TCMs) are mainly used in clinical practice in the form of formulas [1]. To meet the demands of diverse patients, a lot of popular TCM formulas have been developed into patent drugs [2,3,4,5]. However, the quality of these patent drugs may be affected by crude drug materials and manufacturing technologies. For example, the contents of bioactive compounds in different Gegen Qinlian patent drugs varies significantly [6]. Due to their complex chemical composition, clarification of chemical variation of patent drugs derived from the same TCM formula has been a big challenge.

Shengmaisan (SMS) is a popular TCM formula to treat coronary heart diseases and myocardial infarction [7,8,9,10]. It is composed of Hongshen (HS, Ginseng Radix et Rhizoma Rubra), Maidong (MD, Ophiopogonis Radix), and Wuweizi (WWZ, Schisandrae Chinensis Fructus). According to previous reports, SMS contains triterpenoid saponins, steroidal saponins, lignans, etc. [11,12,13,14]. To facilitate use for different patients, SMS has been developed into Shengmai Oral Solution (Shengmaiyin, SMY), Shengmai Capsule (Shengmai Jiaonang, SMJN), Yiqi Fumai Injection (YQFMI), and Yiqi Fumai Capsule (Yiqi Fumai Jiaonang, YQJN). However, their chemical compositions may vary remarkably due to different manufacturing techniques and different proportions of component drugs (Table S1). Currently, schisandrin is the quality control marker of SMY, YQFMI, and YQJN; ginsenosides Rg1 and Re are markers for SMJN; and total ginsenosides are a marker for YQFMI and YQJN [1,15,16]. Thus, it is necessary to clarify the chemical variations among these patent drugs. 

In this study, we developed a three-step strategy to reveal the chemical variations among the four SMS-based patent drugs. Firstly, the chemical constituents in SMS samples were characterized using a UHPLC/Orbitrap-MS. Secondly, untargeted metabolomics was used to discover potential chemical variations in different SMS samples. Finally, the contents of 17 ginsenosides, 3 steroidal saponins, and 5 lignans in 30 batches of SMS-based samples were determined to confirm the chemical variations.

## 2. Results

### 2.1. Chemical Analysis of SMS-Based Patent Drugs

High-resolution mass spectrometry was used to detect and identify the compounds in SMS samples. In total, 227 compounds were tentatively characterized, including 143 ginsenosides, 41 steroidal saponins, and 43 lignans (Table S2). Among them, 36 compounds were identified by comparing with reference standards. 

#### 2.1.1. Identification of Lignans 

**Figure 1:**
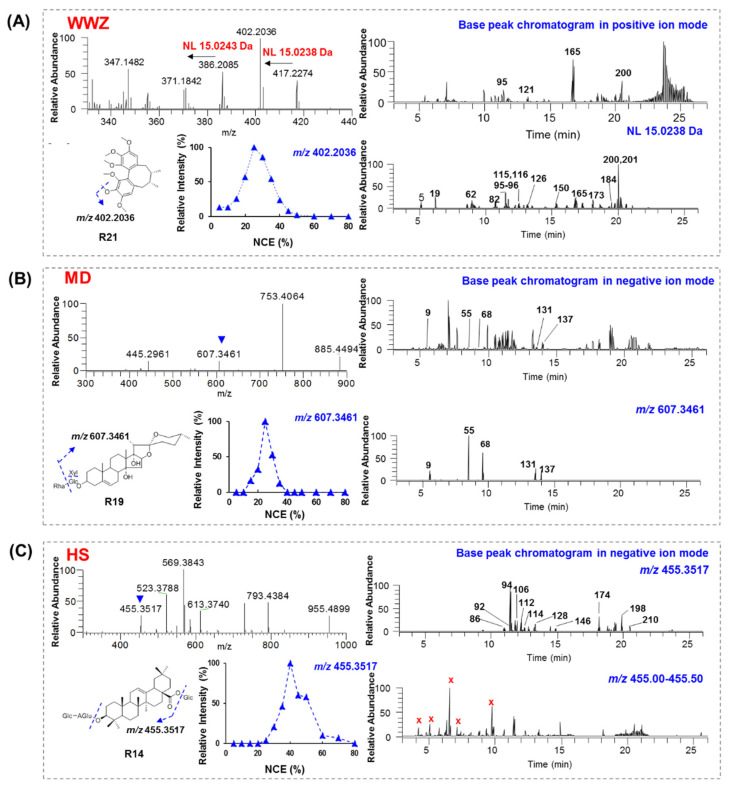
Neutral losses and key product ions were used to identify different types of compounds in SMS. (**A**) The MS/MS spectrum, fragmentation pathway, and optimization of NCE for **R21**, as well as the filtering of NL 15 Da in YQFMI; (**B**) the MS/MS spectrum, fragmentation pathway, and optimization of NCE for **R19**, as well as the filtering of the product ion at *m*/*z* 607.3461 in YQFMI; (**C**) the MS/MS spectrum, fragmentation pathway, and optimization of NCE for **R14**, as well as the filtering of the product ion at *m*/*z* 455.3517 in YQFMI. “X” represents for the false-positive signals.

Lignans are characteristic compounds in WWZ (Figure S1A). The WWZ lignans usually contain methoxyl groups and, thus, could generate a neutral loss (NL) of 15.0238 Da corresponding to a methyl radical (**^.^**CH_3_) in tandem mass spectrometry [17]. Schisandrin (**R21**), one of the main lignans in WWZ, contains six methoxyl groups (Figure 1A). Accordingly, successive NL of 15 Da was observed in its MS/MS spectrum. The normalized collision energy (NCE) of 25% was optimal to yield an abundant product ion at *m*/*z* 402.2036 (NL of 15 Da from the parent ion at *m*/*z* 417.2274). Thus, using the NL of 15.0238 Da as a filter, 38 lignans were detected in the SMS samples (Table S2). Their structures were characterized by comparing them with reference standards or literature data [17,18,19]. For the other 5 lignans (**102**, **183**, **187**, **202**, and **208**) without NL 15 Da in the MS/MS spectra, they were screened using high-resolution MS data and were tentatively identified by comparing them with reported ones [18,19].

#### 2.1.2. Identification of Steroidal Saponins 

Spirostanol-type steroidal saponins are the major compounds in MD [20] (Figure S1B). Two types of key product ions could be observed in their MS/MS spectra. For type I, the key product ion was *m*/*z* 607.3561 (C_33_H_51_O_10_), corresponding to [ophiopogenin-H+C_6_H_10_O_5_]^−^. The optimal NCE was 25% (Figure 1B). For type II, the key product ion was *m*/*z* 575.3592 (C_33_H_51_O_8_), corresponding to [diosgenin-H+C_6_H_10_O_5_]^−^ or [ruscogenin-H+C_6_H_10_O_5_]^−^ (**R25**, Figure S2A). Using these key product ions, compounds containing the same aglycones could be easily screened. As a result, five type I (**9**, **55**, **68**, **131**, **137**) and seven type II (**43**–**47**, **190**, **191**) compounds were rapidly discovered (Figure 1B and Figure S2B). The other 29 steroidal saponins were tentatively identified based on previous reports [14,17,20]. 

#### 2.1.3. Identification of Ginsenosides

Ginsenosides are the major compounds in HS (Figure S1B). 20(*S*)-Protopanaxadiol (PPD), 20(*S*)-protopanaxatriol (PPT), octillol (OT), and oleanolic acid (OA) represent the most common sapogenins for ginsenosides. In the negative ion mode, sequential elimination of the terminal sugar could be observed, and the product ions at *m/z* 455.3517, 459.3844, 475.3799, and 491.3737 were attributed to OA, PPD, PPT, and OT aglycones, respectively (Figure 1C, Figure S3) [21]. For instance, ginsenoside Ro (**R14**) is a typical OA-type ginsenoside and could yield a product ion at *m*/*z* 455.3517 at 40% NCE (Figure 1C). With *m*/*z* 455.3517 as a key product ion, we screened 11 OA-type ginsenosides (**86**, **92**, **94**, **106**, **112**, **114**, **128**, **146**, **174**, **198**, **210**). Meanwhile, high-resolution mass spectral data was also indicated to be essential for reducing false-positive signals. Similarly, 28 PPT-type, 62 PPD-type, and 11 OT-type ginsenosides were also detected (Figure S3) [12,17,21,22,23].

### 2.2. Untargeted Metabolomics to Discover Potential Chemical Variations in SMS Samples

**Figure 2:**
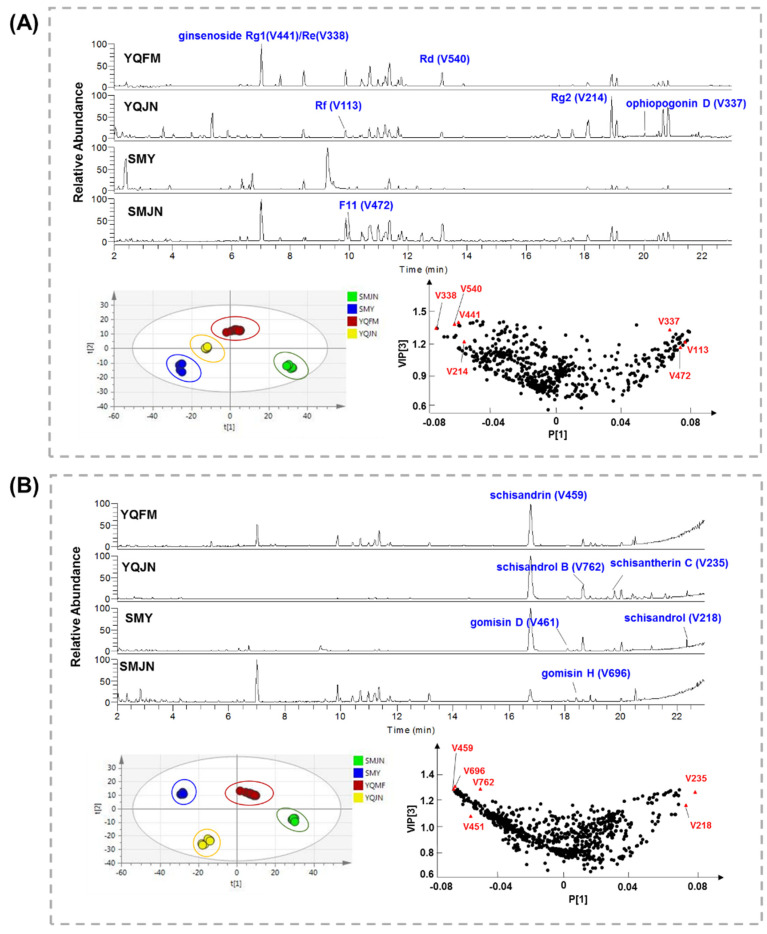
The LC/MS chromatograms of SMS-based patent drugs along with PCA results and variable importance in projection (VIP) plots. (**A**) Negative ion mode; (**B**) positive ion mode.

The second step of this study was to clarify the potential chemical variations using untargeted metabolomics. Both of the negative and positive ion modes were used (Figure 2). The data matrices were generated using the Compound Discoverer software (version 3.1, ThermoFisher). There were 681 and 919 variables in the data matrices in negative and positive ion modes, respectively. Partial least squares discriminant analysis (PLS-DA) was performed using the SIMCA-P software (version 13.0) to figure out important chemical markers to discriminate the four patent drugs (Figure S4). The optimized PLS-DA model for the negative ion mode data described 99.2% of the variations in the response Y (class) (R^2^Y = 0.992), which also predicted 98.8% of the variations (Q^2^ = 0.988). The PLS-DA model for the positive ion mode data described 99.4% of the variations in the response Y (class) (R^2^Y = 0.994), which also predicted 99.2% of the variations (Q^2^ = 0.992). Finally, 13 important variables showing higher intergroup variance with a variable importance in projection (VIP) value >1.1 [24] were identified, including V113, V214, V337, V338, V441, V472, and V540 in the negative ion mode, and V218, V235, V459, V451, V696, and V762 in the positive ion mode (Table S3). Among them, V113, V214, V338, V441, V472, and V540 are ginsenosides; V337 is a steroidal saponin; and V218, V235, V451, V459, V696, and V762 are lignans. These results indicated that ginsenosides, steroidal saponins, and lignans may be the main chemical differences among the four patent drugs. 

### 2.3. Quantitative Analysis of SMS-Based Patent Drugs

**Figure 3:**
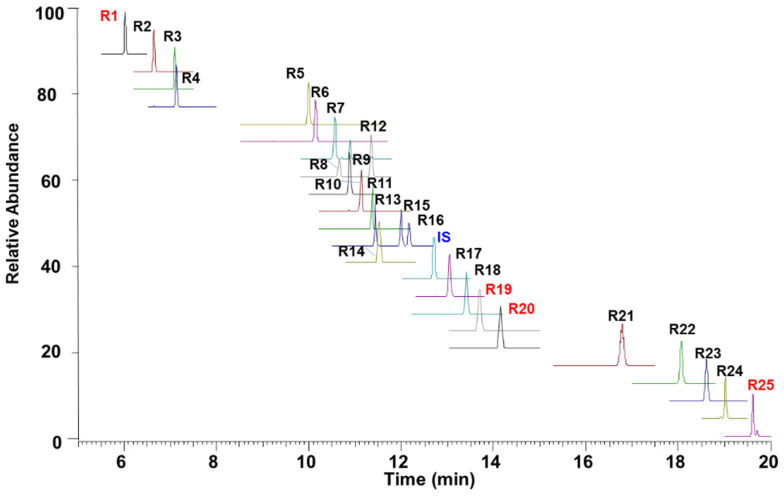
Typical PRM chromatograms of the 25 reference standards. **R1**, **R19**, **R20**, and **R25** were detected in the negative ion mode; **IS** and the other compounds were detected in the positive ion mode.

To further validate the chemical variations among the four SMS-based patent drugs, 25 selected compounds, including 17 ginsenosides, 3 steroidal saponins, and 5 lignans, were quantitatively determined in 30 batches of samples. The typical PRM chromatograms of the mixed reference standards are displayed in Figure 3. The quantitative product ion and collision energy for each analyte was optimized using the MS Tune software (Thermo Scientific) and listed in Table 1.

#### 2.3.1. Method Validation

The calibration curves of 25 analytes were constructed by plotting the analyte/internal standard peak area ratio (Y) against the concentration (X). The internal standard (**IS**, astragaloside IV) was used to guarantee precision of the analyses. All the 25 analytes showed good linearity (*r*^2^ = 0.9925 − 0.9993) (Table S4). The LOQ values ranged from 0.15–156.41 ng/mL (Table 1). The RSD values for intraday and interday precisions ranged from 0.28% to 2.80% and 0.10% to 2.40%, respectively, indicating acceptable precision of the method. The RSD values for the stability analysis ranged from 0.35% to 4.06%. The reproducibility test showed a good consistency of the sample preparation process with RSD values ranging from 0.48%–4.70%. Repeatability, precision, and stability variations are listed in Table S5. Recovery of the analytes varied from 98.43% to 108.10% (Table S6), indicating acceptable accuracy of this method.

#### 2.3.2. Sample Analysis

The validated method was used to analyze 30 batches of SMS-based patent drugs, including 12 batches of YQFMI, 6 batches of YQJN, 6 batches of SMY, and 6 batches of SMJN (Table S7). For samples using schisandrin (**R21**) as a quality control marker, they all met the indicated requirements (0.06 mg/bottle for YQFMI, 0.25 mg/bottle for SMY, 0.15 mg/capsule for YQJN) except for YQJN-1 [5]. On the contrary, the contents of schisandrin in SMJN samples were very low (0.01 mg/capsule), indicating that **R21** should not be used as a marker for SMJN. For ginsenoside Re (**R3**) and ginsenoside Rg1 (**R4**), all the SMJN samples met the requirements (0.45 mg/capsule) and showed low variations (0.85 ± 0.02 mg/capsule). Similarly, the contents of **R3** and **R4** in YQMFI samples were also consistent (0.91 ± 0.15 mg/bottle). However, **R3** and **R4** were hardly detected in SMY and showed great variations in YQJN samples ranging from 0.05 to 1.40 mg/capsule. The total contents of the 25 analytes were consistent in SMJN (3.52 ± 0.06 mg/capsule) and showed moderate variations in SMY and YQFMI (1.30 ± 0.22 and 4.01 ± 0.65 mg/bottle, respectively). For YQJN, significant variations were observed, and the total contents of monitored compounds ranged from 1.45 to 4.30 mg/capsule. The significant intragroup difference among YQJN was mainly due to the variations of the ginsenosides (**R3**, **R4**, **R9**, **R12**, **R13**–**R16**, the total contents ranged from 0.30 to 3.38 mg/capsule) and lignan (**R21**, ranged from 0.01 to 0.61 mg/capsule).

**Figure 4:**
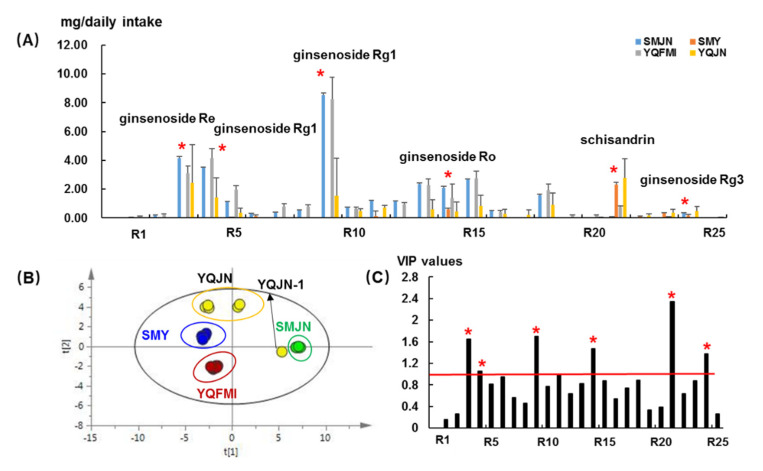
(**A**) The maximum daily intake of 25 compounds when using different SMS-based patent drugs; (**B**) PCA scatter plots for 30 batches of samples; (**C**) VIP values for 25 analytes in different SMS-based patent drugs, *x*-axis represents the first principal component, which explains 62.0% of the total variance, *y*-axis represents the second principal component, which explains 19.7% of the total variance, * represents for the compounds with VIP values > 1.0.

Due to the different packages and medication regimens, we converted the concentrations (mg/capsule or mg/bottle) into a maximum daily dose (YQFMI, 5.20 g/daily dose; YQJN, 2.22 g/daily dose; SMJN, 2.7 g/daily dose; SMY, 30 mL/daily dose) (Table S1). As shown in Figure 4A, when SMJN and YQFMI were used by patients, the daily intake of ginsenosides was much higher than the other patent drugs. The total daily intake of ginsenosides in SMJN was 31.55 ± 0.55 mg/day, 30.80 ± 5.11 mg/day for YQFMI, 11.53 ± 8.75 mg/day for YQJN, and 1.08 ± 0.47 mg/day for SMY. For steroid saponins (**R19**, **R20**, and **R25**), their total daily intake also showed a significant difference, ranging from 0.04 to 0.42 mg/day when using different patent drugs. For lignans (**R21**–**R23**), the total daily intake via YQJN and SMY (3.06 ± 1.16 mg/day) was much higher than SMJN and YQFMI (0.61 ± 0.37 mg/day). 

The results were re-analyzed via principal component analysis (PCA) using the SIMCA-P software. The first and second principal components accounted for 62.0% and 19.7% of the variation, respectively. In Figure 4B, different patent drugs are grouped into separate clusters. YQJN, SMY, and YQFMI appear closer due to their similar chemical contents. The sample YQJN-1 appears apart from the other YQJN samples, probably due to the high content of ginsenosides (25.70 vs. 8.69 mg, YQJN-1 vs. the other YQJN samples). PLS-DA was used to discover the variables contributing to the grouping of these samples (Figure S4). As shown in Figure 4C, the contents of **R3** (ginsenoside Re), **R4** (ginsenoside Rg1), **R9** (ginsenoside Rb1), **R14** (ginsenoside Ro), **R24** (ginsenoside Rg3) from HS, and **R21** (schisandrin) from WWZ showed the highest inter-group variance, as suggested by the largest VIP values (>1.0). Their total contents ranged from 2.81–23.89 mg/daily dose for different patent drugs, which could be the main chemical variations. 

## 3. Materials and Methods

### 3.1. Chemicals and Reagents

**Figure 5:**
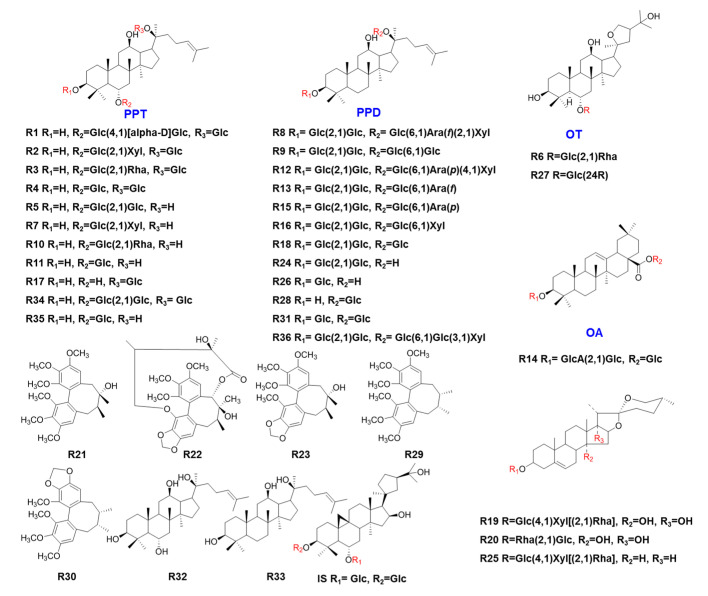
Structures of the 36 reference compounds. PPD, 20(*S*)-protopanaxadiol; PPT, 20(*S*)-protopanaxatriol; OT, octillol; OA, oleanolic acid; glc, glucosyl residue; ara, arabinosyl residue; xyl, xylosyl residue.

The reference standards of notoginsenoside N (**R1**), notoginsenoside R1 (**R2**), ginsenoside Re (**R3**), ginsenoside Rg1 (**R4**), ginsenoside Rf (**R5**), pseudoginsenoside F11 (**R6**), notoginsenoside R2 (**R7**), ginsenoside Ra2 (**R8**), ginsenoside Rb1 (**R9**), ginsenoside Rg2(S) (**R10**), ginsenoside Rh1(S) (**R11**), ginsenoside Ra1 (**R12**), ginsenoside Rc (**R13**), ginsenoside Ro (**R14**), ginsenoside Rb2 (**R15**), ginsenoside Rb3 (**R16**), ginsenoside F1 (**R17**), ginsenoside Rd (**R18**), ginsenoside Rg3 (**R24**), ginsenoside Rh2 (**R26**), pseudoginsenoside RT5 (**R27**), ginsenoside CK (**R28**), schisandrin A (**R29**), schisandrin B (**R30**), ginsenoside f2 (**R31**), protopanaxatriol (**R32**), protopanaxadiol (**R33**), 20-*O*-glucosylginsenoside Rf (**R34**), ginsenoside Rh1(R) (**R35**), and ginsenoside Ra3 (**R36**) were isolated by the authors’ group from Ginseng Radix et Rhizoma, and their structures were identified via NMR analysis [21,22]. Ophiopogonin C (**R19**), opennogenin 3-*O*-α-l-rhamnopyranosyl-(1-2)-β-d-glucopyranoside (**R20**), schisandrin (**R21**), gomisin D (**R22**), schisandrol B (**R23**), ophiopogonin D (**R25**), and astragaloside IV (**IS**, internal standard) were purchased from Chengdu DeSiTe Biological Technology Co., Ltd. (Chengdu, China). Their structures are shown in Figure 5. Their purities were >98% via HPLC analysis. HPLC grade methanol, acetonitrile, and formic acid were obtained from Fisher Scientific (Fair Lawn, NJ, USA). De-ionized water was prepared by using the Milli-Q purification system (Millipore, Billerica, MA, USA).

Hongshen (HS, Ginseng Radix et Rhizoma Rubra), Maidong (MD, Ophiopogonis Radix), and Wuweizi (WWZ, Schisandrae Chinensis Fructus) extracts and YQFMI 1–12 (0.65 g/daily dose) were kindly donated by company ***a***. YQJN 13–18 (2.22 g/daily dose) were supplied by company ***b***. SMJN 19–24 (2.7 g/daily dose) and SMY 25–30 (30 mL/daily dose) were obtained from companies ***c*** and ***d***, respectively. Detailed information of the samples is listed in Table S6. Voucher specimens were deposited at the School of Pharmaceutical Sciences, Peking University (Beijing, China).

### 3.2. Sample Solution Preparation

#### 3.2.1. Preparation of Reference Standard Solutions

For qualitative analysis and untargeted metabolomics, an appropriate amount of the 36 reference standards was dissolved in methanol to prepare a mixed standard solution (10 μg/mL for each compound). For quantitative analysis, a mixed stock solution (**R1**–**R25**) was prepared by dissolving an appropriate amount of each reference standard in methanol. The mixed standard solution was then serially diluted (dilution factor = 4/3, 2, 4, 8, 32, 128, 512, 2048, 8192, 32768, 131072, and 524288) using methanol. The series of calibration solutions were then diluted by 2-fold using the internal standard solution (IS, astragaloside IV, 500 ng/mL), respectively. 

#### 3.2.2. Preparation of Sample Solutions

For the qualitative and untargeted metabolomic analysis, 100 mg of YQFMI, YQJN, and SMJN as well as 1.0 mL of SMY were respectively dissolved in 2 mL of solvent (10% methanol). In addition, 100 mg of HS, MD, and WWZ extracts were individually dissolved in 2 mL of solvent (10% methanol). For quantitative analysis, 100 mg of YQFMI, YQJN, and SMJN as well as 1.0 mL of SMY were respectively diluted with 50 mL of methanol. The samples for LC/MS analysis were then diluted by 2-fold using the IS solution.

### 3.3. Liquid Chromatography

A Vanquish UHPLC system (Thermo Fisher Scientific Inc., Waltham, MA, USA) was used for liquid chromatography. Samples were separated on an Acquity UPLC HSS T3 column (100 mm × 2.1 mm, 1.8 µm) equipped with a VanGuard pre-column (5 mm × 2.1 mm, 1.8 µm) (Waters, Milford, MA, USA). The mobile phase A was water containing 0.1% formic acid and B was acetonitrile. The gradient elution program was set as follows: 0–4 min, 10–25% B; 4–8 min, 25–35% B; 8–16 min, 35–45% B; 16–20 min, 45–75% B; 20–22 min, 75–95% B; 22–24 min, 95% B. The flow rate was 300 μL/min and the column temperature was set at 40 °C. The injection volume was 2 μL.

### 3.4. Mass Spectrometry 

Mass spectrometry analysis was performed on a Q-Exactive hybrid quadrupole Orbitrap mass spectrometer (Thermo Scientific, San Jose, CA, USA) equipped with a heated electrospray ionization source (HESI). It was operated in both negative and positive ion modes. The other parameters were set as follows: spray voltage, ±3.5 kV; sheath gas flow rate, 45 arb; auxiliary gas, 10 arb; capillary temperature, 350 °C; auxiliary temperature, 350 °C; S-lens RF level, 60 V. Full Scan/dd-MS^2^ was used to acquire the qualitative and untargeted metabolomics data. The resolutions for MS and MS/MS were set as 70,000 and 17,500, respectively. The scan range was set as *m*/*z* 100–1500, and the normalized collision energies (NCE) were 25% and 40%. The ten most abundant ions in each full scan were selected as precursor ions to obtain their MS/MS spectra. Parallel reaction monitoring (PRM) mode was used to acquire the targeted data. Polarity switch was used to detect the 25 selected compounds in a single run. The optimized NCEs and other quantitative information about the 25 reference standards are shown in Table 1. Data were processed using the Xcalibur^TM^ 4.1 software (ThermoFisher, San Jose, CA, USA, 2019).

### 3.5. Method Validation

A mixed standard solution (1250 ng/mL for each analyte) was used for precision and stability tests. Intra- and interday precision was assessed by testing a sample solution six times a day for three consecutive days. The stability was evaluated by analyzing the same solution at 0, 2, 4, 8, 12, and 24 h at 15 °C. The repeatability was described by analyzing six samples (YQFMI-3) prepared using the same method. Accuracy was evaluated by standard addition tests, where a prepared YQFMI-3 sample was mixed with reference standards at the same level (around 100% of the contents, *n* = 6). Recoveries were calculated using the formula: recovery (%) = (found amount—original amount)/spiked amount × 100%. The limit of quantitation (LOQ) was set at the lowest concentration of the calibration curves according to requirements set by the US Food and Drug Administration [25]. The details are listed in Table 1 and Table S3.

## 4. Conclusions

In this study, an integrated strategy was proposed to reveal the chemical variations among four SMS-based patent drugs. Firstly, 227 compounds were identified using a UHPLC/Orbitrap-MS. Secondly, untargeted metabolomics revealed that ginsenosides, steroid saponins, and lignans were significantly different for the SMS-based samples. Finally, the contents of 25 compounds in 30 batches of samples were determined to confirm the chemical variations. Ginsenosides Re, Rg1, Rb1, Ro, and Rg3 as well as schisandrin were the main chemical markers to differentiate the four SMS-based patent drugs. 

## Figures and Tables

**Table 1:** Precursor/product ion pairs and PRM parameters of the 25 analytes used in this study.

Analyte	Compound Name	Formula	Retention Time (min)	Type	PRM Transition Precursor Ion → Product Ion (*m*/*z*)	Ion Mode	NCE
**R1**	Notoginsenoside N	C_48_H_82_O_19_	6.03	+ HCOO	1007.5432 → 475.38	Negative	31
**R2**	Notoginsenoside R_1_	C_47_H_80_O_18_	6.64	+ Na	955.5237 → 775.46	Positive	40
**R3**	Ginsenoside Re	C_48_H_82_O_18_	7.10	+ Na	969.5393 → 789.48	Positive	29
**R4**	Ginsenoside Rg_1_	C_42_H_72_O_14_	7.14	+ Na	823.4814 → 643.42	Positive	27
**R5**	Ginsenoside R*_f_*	C_42_H_72_O_14_	9.99	+ Na	823.4814 → 365.11	Positive	37
**R6**	Pseudoginsenoside F_11_	C_42_H_72_O_14_	10.15	+ Na	823.4814 → 497.36	Positive	37
**R7**	Notoginsenoside R_2_	C_41_H_70_O_13_	10.55	+ Na	793.4709 → 335.10	Positive	35
**R8**	Ginsenoside Ra_2_	C_58_H_98_O_26_	10.66	+ Na	1233.6239 → 467.14	Positive	32
**R9**	Ginsenoside Rb_1_	C_54_H_92_O_23_	10.89	+ Na	1131.5922 → 365.11	Positive	31
**R10**	Ginsenoside Rg_2_(*S*)	C_42_H_72_O_13_	11.13	+ Na	807.4865 → 349.11	Positive	33
**R11**	Ginsenoside Rh_1_(*S*)	C_36_H_62_O_9_	11.37	+ Na	661.4286 → 481.37	Positive	35
**R12**	Ginsenoside Ra_1_	C_58_H_98_O_26_	11.36	+ Na	1233.0000 → 467.14	Positive	32
**R13**	Ginsenoside Rc	C_53_H_90_O_22_	11.46	+ Na	1101.5816 → 335.25	Positive	33
**R14**	Ginsenoside Ro	C_48_H_76_O_19_	11.54	+ Na	979.4873 → 361.07	Positive	29
**R15**	Ginsenoside Rb_2_	C_53_H_90_O_22_	12.01	+ Na	1101.5816 → 335.09	Positive	33
**R16**	Ginsenoside Rb_3_	C_53_H_90_O_22_	12.17	+ Na	1101.5816 → 335.09	Positive	33
**R17**	Ginsenoside f_1_	C_36_H_62_O_9_	13.05	+ Na	661.4286 → 481.36	Positive	29
**R18**	Ginsenoside Rd	C_48_H_82_O_18_	13.43	+ Na	969.5393 → 789.47	Positive	30
**R19**	Ophiopogonin C	C_44_H_70_O_18_	13.70	+ HCOO	931.4544 → 753.41	Negative	25
**R20**	Opennogenin-3-*O*-α-l-Rhamnopyranosyl-(1-2)-β-d-Glucopyranoside	C_39_H_62_O_14_	14.15	+ HCOO	799.4122 → 753.41	Negative	25
**R21**	Schisandrin	C_24_H_32_O_7_	16.80	+ H	433.2221 → 384.19	Positive	23
**R22**	Gomisin D	C_28_H_34_O_10_	18.07	+ Na	553.2044 → 507.20	Positive	28
**R23**	Schisandrol B	C_23_H_28_O_7_	18.63	-H	399.1802 → 369.17	Negative	30
**R24**	Ginsenoside Rg_3_	C_42_H_72_O_13_	19.02	+ Na	807.4865 → 365.10	Positive	38
**R25**	Ophiopogonin D	C_44_H_70_O_16_	19.62	+ HCOO	899.4646 → 721.42	Negative	25
**IS**	Astragaloside IV	C_41_H_68_O_14_	12.72	+ Na	807.4501 → 627.39	Positive	39

## Data Availability

Not applicable.

## Supplementary Materials

The following are available online. Figure S1: UHPLC/Q-Orbitrap MS chromatograms of YQFMI and WWZ in positive ion mode (A) and UHPLC/Q-Orbitrap MS chromatograms of YQFMI, HS, and MD in negative ion mode (B). Figure S2: LC/MS chromatograms of YQFMI using key product ion at *m*/*z* 575.3592 for Type II steroidal saponins from MD. (A): MS/MS spectrum of R25, (B): the filtering result of product ion at *m*/*z* 575.3592 in YQFM, and (C) the fragment pathway of R25. Figure S3: LC/MS chromatograms of YQFMI using different key product ions for ginsenosides from HS. (A) for PPT type ginsenosides using product ion at *m*/*z* 475.3789; (B) for PPD type ginsenosides using product ion at *m/z* 459.3854; (C) for OT type ginsenosides using product ion at *m*/*z* 491.3745. Figure S4: The established PLS-DA model for different SMS-based patent drugs. (A) PLS-DA plots; (B) permutation test, the y-axis represents the frequency of accuracy of 200 models in the 200 Permutation Test, and the x-axis represents the location of the accuracy of the PLS-DA model, R2 represents the interpretation rate of the established model, and Q2 represents the predictive power of the model; (C) CV-ANOVA test. Table S1: The components of different SMS-based patent drugs. Table S2: Characterization of the chemical constituents in SMS using UHPLC/orbitrap-MS. Table S3: Information of 13 important variables showed higher inter-group variance. Table S4: Linear regression data of the 25 analytes. Table S5: Repeatability, precision and stability variations of 25 analytes. Table S6: Recovery of the analytes (n = 6). Table S7: The contents of 25 analytes in 30 batches of SMS-based patent drugs. Table S8: Detailed information for the 30 batches of different SMS-based patent drugs.

## Sample Availability

Samples of the YQFMI are available from Tianjin Tasly Pharmaceutical Co. Ltd.
