# Supplementary material for: Chemical Variations among Shengmaisan-Based TCM Patent Drugs by Ultra-High Performance Liquid Chromatography Coupled with Hybrid Quadrupole Orbitrap Mass Spectrometry
Source: Molecules. 2021 Jun 30;26(13):4000. doi: 10.3390/molecules26134000 (PMC8271701; doi:10.3390/molecules26134000)
Supplement: Supplementary file 1 [file molecules-26-04000-s001.zip › molecules-1208108-supplementary.pdf]

Chemical variations among Shengmaisan-based TCM patent drugs by ultra-high performance liquid chromatography cou-pled with hybrid quadrupole Orbitrap mass spectrometry

Lulu Xu[1,2,†], Zhanpeng Shang[1,†], Yungang Tian[1], Ming Xiong[1], Dilaram Nijat[1], Yuan Wang[1], Xue Qiao[1,4*], Min Ye[1,3,4*]

Affiliations

[1] *State Key Laboratory of Natural and Biomimetic Drugs, School of Pharmaceutical Sciences, Peking University, 38 Xueyuan Road, Beijing 100191, China*

[2] *School of Chinese Materia Medica, Beijing University of Chinese Medicine, Beijing 100029, China*

[3] *Key Laboratory of Molecular Cardiovascular Sciences of Ministry of Education, Peking University, Beijing 100191, China*

[4] *Department of Integration of Chinese and Western Medicine, School of Basic Medical Sciences, Peking University, Beijing 100191, China*

[*] Corresponding authors. Tel.: +86 10 82801516. Fax: +86 10 82802024. Email address: qiaoxue@bjmu.edu.cn (X. Qiao) or yemin@bjmu.edu.cn (M. Ye).

† These authors contributed equally to this work.

Table of Contents

Figure S1. UHPLC/Q-Orbitrap MS chromatograms of YQFMI and WWZ in positive ion mode (A) and UHPLC/Q-Orbitrap MS chromatograms of YQFMI, HS, and MD in negative ion mode (B).

Figure S2. LC/MS chromatograms of YQFMI using key product ion at *m/z* 575.3592 for Type II steroidal saponins from MD. (A): MS/MS spectrum of **R25**, (B): the filtering result of product ion at *m/z* 575.3592 in YQFM, and (C) the fragment pathway of **R25**.

Figure S3. LC/MS chromatograms of YQFMI using different key product ions for ginsenosides from HS. (A) for PPT type ginsenosides using product ion at *m/z* 475.3789; (B) for PPD type ginsenosides using product ion at *m/z* 459.3854; (C) for OT type ginsenosides using product ion at *m/z* 491.3745.

Figure S4. The established PLS-DA model for different SMS-based patent drugs. (A) PLS-DA plots; (B) permutation test, the y-axis represents the frequency of accuracy of 200 models in the 200 Permutation Test, and the x-axis represents the location of the accuracy of the PLS-DA model, R2 represents the interpretation rate of the established model, and Q2 represents the predictive power of the model; (C) CV-ANOVA test.

Table S1. The components of different SMS-based patent drugs.

Table S2. Characterization of the chemical constituents in SMS using

UHPLC/orbitrap-MS.

Table S3. Information of 13 important variables showed higher inter-group variance.

Table S4. Linear regression data of the 25 analytes.

Table S5. Repeatability, precision and stability variations of 25 analytes.

Table S6. Recovery of the analytes (*n* = 6).

Table S7. The contents of 25 analytes in 30 batches of SMS-based patent drugs.

Table S8. Detailed information for the 30 batches of different SMS-based patent drugs.

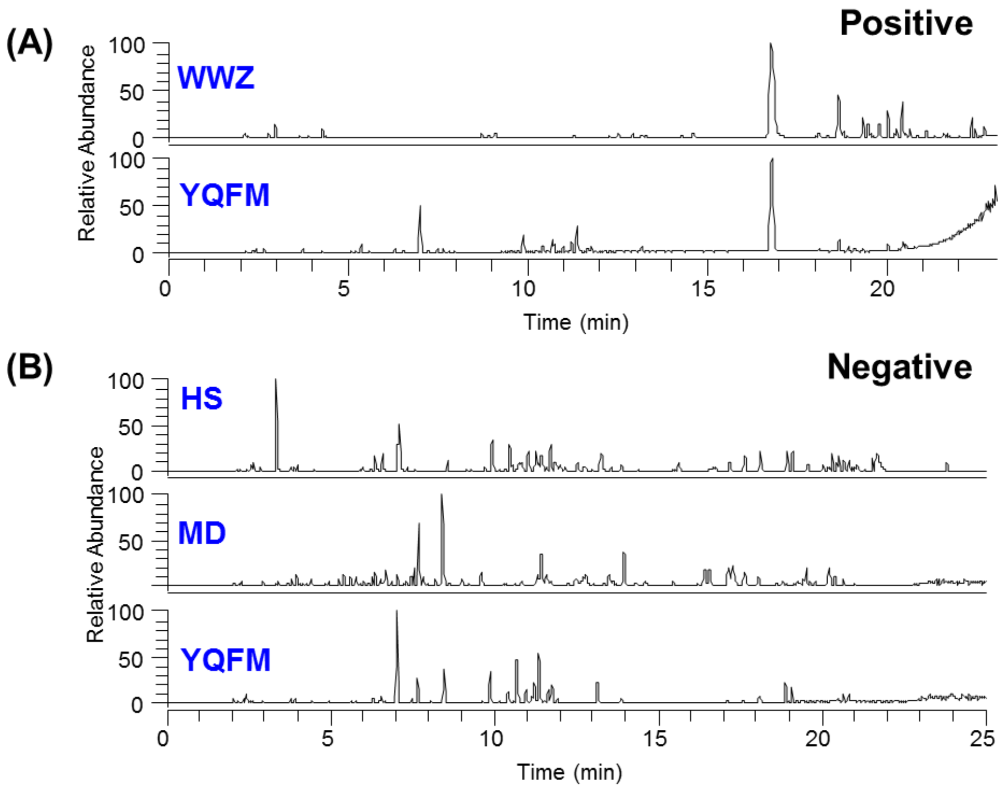

Figure S1. UHPLC/Q-Orbitrap-MS chromatograms of YQFMI and WWZ in positive ion mode (A) and UHPLC/Q-Orbitrap-MS chromatograms of YQFMI, HS, and MD in negative ion mode (B).

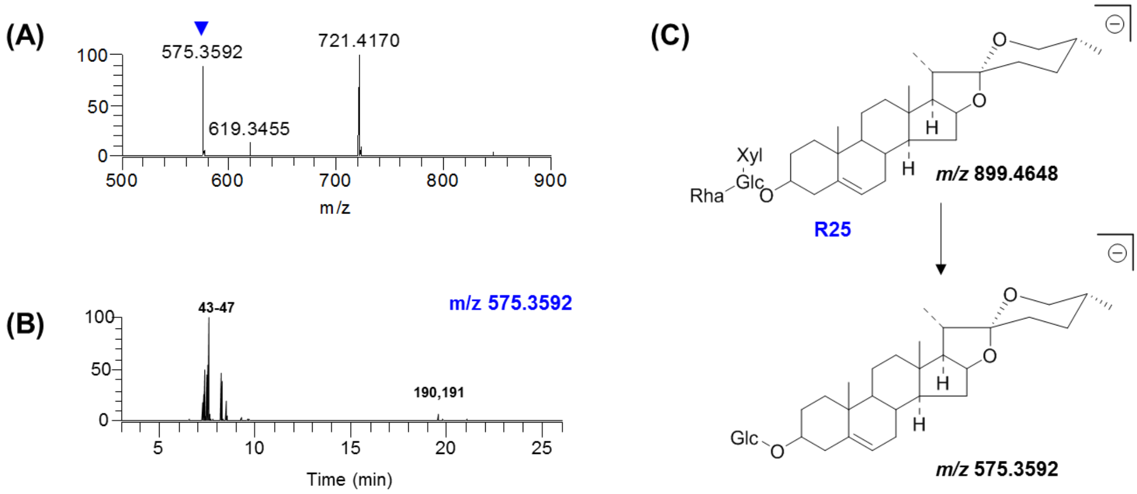

Figure S2. LC/MS chromatograms of YQFMI using key product ion at *m/z* 575.3592 for Type II steroidal saponins from MD. (A): MS/MS spectrum of **R25**, (B): the filtering result of product ion at *m/z* 575.3592 in YQFM, and (C) the fragment pathway of **R25**.

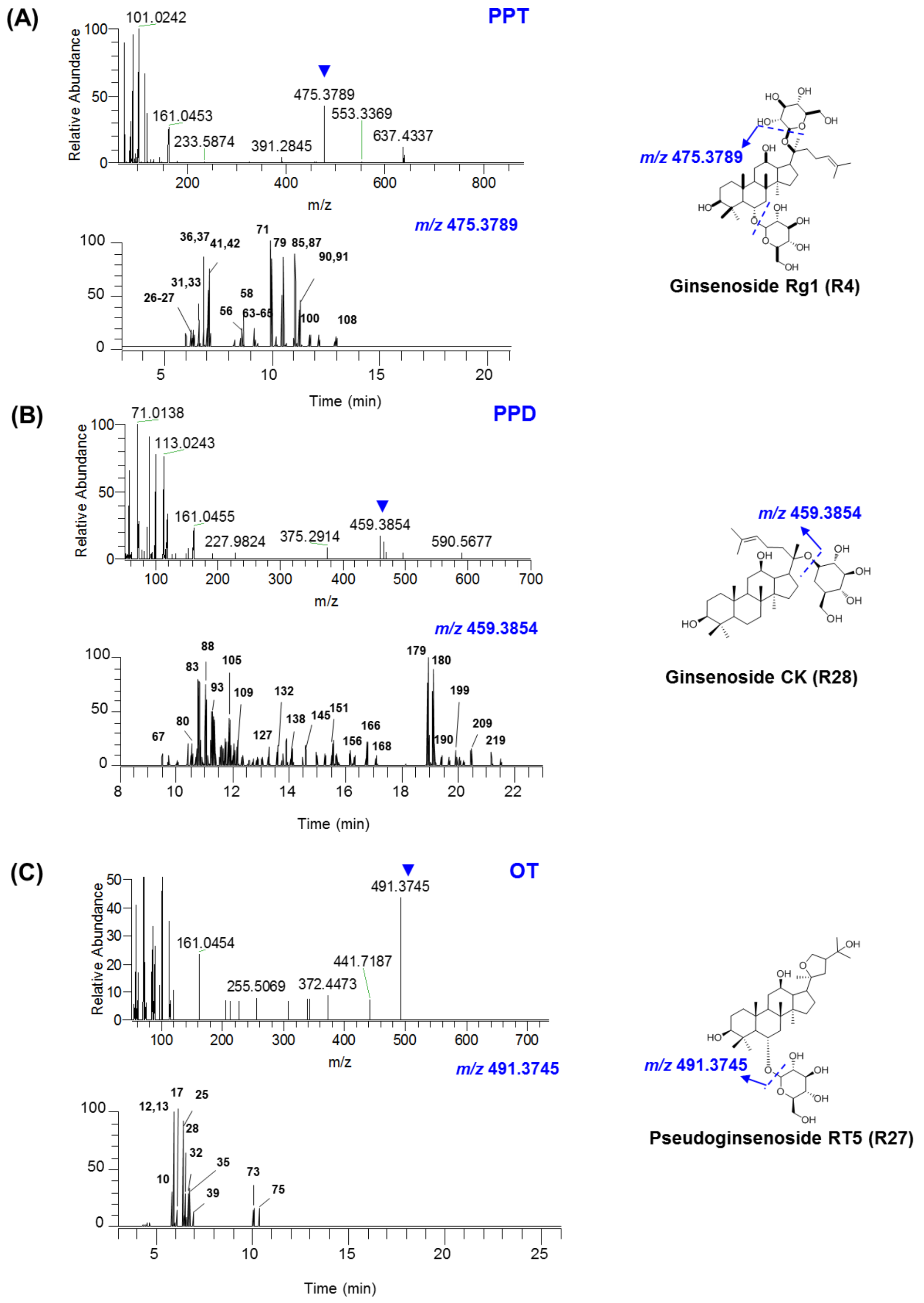

Figure S3. LC/MS chromatograms of YQFMI using different key product ions for ginsenosides from HS. (A) for PPT type ginsenosides using product ion at *m/z* 475.3789; (B) for PPD type ginsenosides using product ion at *m/z* 459.3854; (C) for OT type ginsenosides using product ion at *m/z* 491.3745.

(A)

(B)

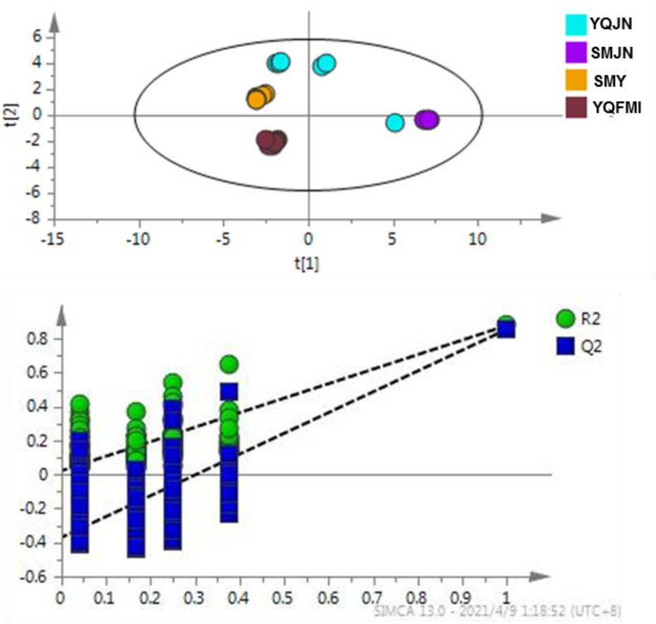

(C)

	1	2	3	4	5	6	7
1	M3(Untitled)	SS	DF	MS	F	p	SD
2	Total corr.	87	87	1			1
3	Regression	80.0014	24	3.33339	30.0067	6.78234e-026	1.82576
4	Residual	6.99856	63	0.111088			0.333299

Figure S4. The established PLS-DA model for different SMS-based patent drugs. (A) PLS-DA plots; (B) permutation test, the y-axis represents the frequency of accuracy of 200 models in the 200 Permutation Test, and the x-axis represents the location of the accuracy of the PLS-DA model, R2 represents the interpretation rate of the established model, and Q2 represents the predictive power of the model; (C) CV-ANOVA test.

Table S1. The components of different SMS-based patent drugs.

Formula	Components	Proportion	Specification	Maximum Daily dosage	Quality criterion
SMY	HS, MD, WWZ	1:2:1	10 mL	30 mL	Schisandrin, ≥0.25 mg/10mL
SMJN	HS, MD, WWZ	1:2:1	0.30 g	2.70 g	Ginsenoside Rg1+Ginsenoside Re ≥0.45 mg/0.3 g
YQFMI	HS, MD, WWZ	1:3:1.5	0.65 g	5.2 g	Schisandrin, ≥0.06 mg/0.65 g; Total ginsenosides ≥5 mg/0.65 g
YQJN	HS, MD, WWZ	Not accessible	0.37 g	2.96 g	Schisandrin, ≥0.15 mg/0.37 g; Total ginsenosides ≥5 mg/0.37 g

Note: SMY, Shengmaiyin; SMJN, Shengmai Jiaonang; YQFMI, Yiqi Fumai Injection; YQJN, Yiqi Fumai Jiaonang. HS, Hongshen; MD, Maidong; WWZ, Wuweizi.

Table S2. Characterization of the chemical constituents in SMS using UHPLC/orbitrap-MS.

No.	t_R/min	Molecular formula	(*m/z*) predicted	[M−H]⁻ measured	Δ (ppm)	MS/MS (*m/z*)	Identification	Type	Source	a	b	c	d
1	4.19	$C_{41}H_{70}O_{15}$	847.4697	847.4699	1.5	649.4213,451.4118,379.0791	Floralginsenoside D		HS	√	√		
2	4.75	$C_{42}H_{72}O_{16}$	877.4802	877.4806	1.65	663.3713,553.3308,391.2850	Floralginsenoside B		HS	√			
3	4.92	$C_{36}H_{62}O_{11}$	715.4274	715.4277	1.89	593.3711,449.3270,431.3167,391.2852	Floralginsenoside Ka		HS	√			
4	4.97	$C_{36}H_{62}O_{11}$	715.4274	715.4278	2.07	593.3667,449.3259,431.3154,373.0761	Ginsenoside SL1		HS	√			
5#	5.12	$C_{22}H_{26}O_6$	387.1802	387.1798	-1.12	372.1561,357.1285,355.1534,342.1395,327.1590,323.1273	Gomisin M1 isomer		WWZ	√		√	
6	5.17	$C_{36}H_{62}O_{11}$	715.4274	715.4274	1.47	593.3684,449.3282,431.3158	Ginsenoside Rh6		HS	√			
7	5.32	$C_{50}H_{82}O_{22}$	1079.5279	1079.5277	0.77	917.4732,771.4170,609.3659,447.3129	Parisaponin I isomer		MD	√			
8	5.43	$C_{50}H_{82}O_{22}$	1079.5279	1079.5271	0.2	917.4735,771.4168,609.3645,447.3129	Parisaponin I isomer		MD	√			
9	5.56	$C_{46}H_{76}O_{22}$	979.4744	979.4757	1.35	933.4711,787.4113,787.4113,607.3479	/		MD				
10	5.81	$C_{43}H_{74}O_{17}$	861.4842	861.4853	1.12	815.4774,653.4236,491.3737,391.2851	/	OT	HS				
11	5.86	$C_{50}H_{82}O_{22}$	1079.5279	1079.5275	0.55	899.4656,753.4057,591.3530,429.2989	Parisaponin I isomer		MD	√			
12	5.91	$C_{36}H_{62}O_{10}$	861.4842	861.4854	1.18	815.4812,653.4271,491.3739,391.2851	/	OT	HS				
13	5.91	$C_{48}H_{68}O_{13}$	851.4576	851.4566	-0.94	491.3736,391.2850	/	OT	HS				
14*	5.99	$C_{48}H_{82}O_{19}$	1007.5432	1007.5436	1.43	961.5453,637.4325,619.4217,475.3794	Notoginsenoside N*	PPT	HS	√	√		
15	6	$C_{45}H_{74}O_{19}$	917.4752	917.4742	0.14	771.4171,609.3657,447.3115	Ruscogenin 1-O-α-L-Xyl(1→3)Rha(1→2)Ara isomer		MD	√			√
16	6.02	$C_{50}H_{82}O_{22}$	1079.5279	1079.5271	0.2	899.4717,735.3999,555.3320,467.2130	Parisaponin I isomer		MD	√			
17	6.05	$C_{43}H_{74}O_{17}$	861.4842	861.4848	0.63	815.4756,653.4266, 491.3737	/	OT	HS				
18	6.19	$C_{50}H_{82}O_{22}$	1079.5279	1079.5271	0.2	917.4737,771.4171,591.3531,429.3019	Parisaponin I isomer		MD	√			

19#	6.2	$C_{22}H_{26}O_6$	387.1802	387.1796	-1.51	372.1559,357.1272,355.1534,340.1293,327.1589,325.1074,323.1272	Gomisin M1		WWZ	√		√	
20	6.24	$C_{47}H_{80}O_{18}$	977.5327	977.5327	1.1	637.4317,553.3386,475.3786	Notoginsenoside R1 isomer	PPT	HS	√			
21	6.27	$C_{18}H_{34}O_{10}$	455.2134	455.2133	2.1	411.2013	Isoheptanol 2(S)-*O*-*β*-D-xylopyranosyl-(1→6)-*O*-*β*-D-glucopyranoside isomer		HS	√		√	
22	6.33	$C_{50}H_{82}O_{22}$	1079.5279	1079.527	0.09	917.4722,771.4169,609.3646,429.3003	Parisaponin I		MD	√			
23*	6.34	$C_{48}H_{82}O_{19}$	1007.5432	1007.5429	0.71	637.4315,619.4201,475.3788	20-*O*-glucosylginsenoside Rf*	PPT	HS	√	√		
24	6.36	$C_{18}H_{34}O_{10}$	455.2134	455.213	1.55	409.2077	Isoheptanol 2(S)-*O*-*β*-D-xylopyranosyl-(1→6)-*O*-*β*-D-glucopyranoside isomer		HS	√		√	√
25	6.39	$C_{42}H_{72}O_{16}$	831.4736	831.4745	0.88	786.4707,653.4290,553.3407,491.3739,391.2851	/	OT	HS	√			
26	6.43	$C_{47}H_{80}O_{18}$	977.5327	977.5326	1.04	637.4288,475.3783	Notoginsenoside R1 isomer	PPT	HS	√			
27	6.47	$C_{48}H_{82}O_{19}$	1007.5432	1007.5432	1.07	961.5504,796.4082,637.4317,475.3786	Notoginsenoside N isomer	PPT	HS	√			
28	6.5	$C_{36}H_{62}O_{10}$	699.4325	699.4323	1.3	491.3737,447.3121,403.3214,391.2868	Pseudoginsenoside RT5 isomer	OT	HS	√			
29	6.57	$C_{50}H_{82}O_{22}$	1079.5279	1079.526	-0.82	917.4765,771.4161,609.3653,447.3138	Parisaponin I isomer		MD	√		√	
30	6.59	$C_{18}H_{34}O_{10}$	455.2134	455.2128	1.09	409.2075	Isoheptanol 2(S)-*O*-*β*-D-xylopyranosyl-(1→6)-*O*-*β*-D-glucopyranoside		HS	√	√	√	√
31*	6.59	$C_{47}H_{80}O_{18}$	977.5327	977.532	0.48	769.4779,637.4311,475.3785	Notoginsenoside R1*	PPT	HS	√			√
32	6.64	$C_{36}H_{62}O_{10}$	699.4325	699.4321	0.95	553.3374,491.3732,391.2852	Pseudoginsenoside RT5 isomer	OT	HS	√			
33	6.64	$C_{48}H_{82}O_{19}$	1007.5432	1007.5432	1.01	961.5324,799.4863,637.4318,475.3787	Notoginsenoside N isomer	PPT	HS	√			
34	6.66	$C_{50}H_{82}O_{22}$	1079.5279	1079.5272	0.32	917.4759,771.4168,609.3632,447.3125	Parisaponin I isomer		MD	√			
35	6.71	$C_{43}H_{74}O_{16}$	845.4893	845.49	0.73	799.4862,653.4281,491.3738,391.2850	/	OT	HS				
36	6.79	$C_{47}H_{80}O_{18}$	977.5327	977.5324	0.86	637.4318,475.3789	Notoginsenoside R1 isomer	PPT	HS	√			

37	6.8	$C_{48}H_{82}O_{19}$	1007.5432	1007.5431	0.95	637.4324,475.3790	20-*O*-glucosylginsenoside R*f* isomer	PPT	HS	√			
38	6.88	$C_{45}H_{74}O_{19}$	917.4752	917.4747	0.73	611.3057,305.1598	Ruscogenin 1-O-α-L-Xyl(1→3)Rha(1→2)Ara isomer		MD	√			√
39	6.91	$C_{42}H_{72}O_{1}$	861.4842	861.4851	0.94	815.4798,653.4266,491.3775	/	OT	HS				
40	7.04	$C_{45}H_{74}O_{19}$	917.4752	917.473	-1.2	771.4187,609.3648,591.3531,429.3018	Ruscogenin 1-O-α-L-Xyl(1→3)Rha(1→2)Ara		MD	√			√
41*	7.05	$C_{48}H_{82}O_{18}$	991.5483	991.5474	0.2	945.5438, 637.4317,475.3789	Ginsenoside Re*	PPT	HS	√	√		
42*	7.09	$C_{42}H_{72}O_{14}$	845.4904	845.4899	0.66	637.4336,553.3368,475.3788,457.3728,391.2845	Ginsenoside Rg1*	PPT	HS	√	√		√
43	7.25	$C_{57}H_{94}O_{29}$	1241.5797	1241.5797	0.01	1195.5709,1063.5366,901.4788,755.4219,575.3591	/		MD				
44	7.25	$C_{42}H_{70}O_{12}$	643.2901	643.289	-1.7	593.3676,581.2244,575.3593	/		MD				
45	7.34	$C_{38}H_{44}O_{9}$	643.2901	643.289	-1.7	593.3694,575.3580,531.2633	/		MD				
46	7.34	$C_{56}H_{92}O_{27}$	1195.5742	1195.5741	-0.1	1063.5293,901.4801,755.4217,575.3581	/		MD				
47	7.34	$C_{25}H_{49}O_{17}$	620.2886	620.286	-4.17	593.3684,575.3584	/		MD				
48	7.7	$C_{21}H_{36}O_{10}$	493.229	493.2287	1.52	447.2232,382.5669,315.1811,359.1698	Bornyl 7-*O*-*α*-L-arabinofuranosyl-(1→6)-*β*-D -glucopyranoside		MD	√	√	√	√
49	7.9	$C_{44}H_{70}O_{16}$	899.4643	899.4641	0.62	735.3939,573.3378	Ophiopogonin D isomer		MD	√			
50	7.93	$C_{53}H_{90}O_{23}$	1139.5855	1139.5863	1.67	799.4840,781.4748,619.4190,537.3442	Quinquenoside L7		HS	√			
51	8.1	$C_{21}H_{36}O_{10}$	493.229	493.2289	1.88	447.2232	L-borneol-7-*O*-[*β*-D-apiofuranosyl (1→6)]-*β*-D-glucopy-ranoside isomer		HS	√			
52	8.11	$C_{53}H_{90}O_{23}$	1139.5855	1139.5856	1.03	781.4749,619.4209,537.3460	Floralnotoginsenoside A		HS	√			
53	8.22	$C_{53}H_{90}O_{23}$	1139.5855	1139.585	0.5	799.4813,637.4306,475.3799	Floralnotoginsenoside D	PPT	HS	√			
54	8.24	$C_{50}H_{82}O_{22}$	1079.5279	1079.5278	0.88	901.4792,755.4217,593.3693,431.3164	Ophiopogoside A		MD	√			
55	8.48	$C_{50}H_{80}O_{24}$	1063.4955	1063.4963	0.71	885.4492,739.3905,607.3485	/		MD				

56	8.58	$C_{44}H_{74}O_{15}$	887.501	887.5013	1.65	637.4352，619.4210,475.3761	6'-Acetyl-ginsenoside Rg 1	PPT	HS	√	√		
57#	8.6	$C_{22}H_{26}O_6$	387.1802	387.1801	-0.27	372.1582,357.1627,355.1534,342.1413,337.1431,325.1416,324.1351,310.1195	Gomisin M1 isomer		WWZ	√		√	√
58	8.69	$C_{53}H_{90}O_{23}$	1139.5855	1139.585	0.5	1093.5626, 799.4830,637.4323,475.3800	Gyponoside LXXI	PPT	HS	√			
59#	8.93	$C_{23}H_{28}O_6$	401.1959	401.1956	-0.61	386.1721,370.1766,355.1536,338.1506,332.1245	Gomisin N isomer		WWZ	√			
60	9.03	$C_{44}H_{70}O_{16}$	899.4643	899.4641	0.62	573.3491	Ophiopogonin D isomer		MD	√			
61	9.05	$C_{45}H_{72}O_{19}$	927.4595	927.46	1.74	881.4516,735.3995,573.3450	Ac-Ophiopojaponin C		MD	√	√	√	√
62#	9.07	$C_{22}H_{26}O_6$	387.1802	387.18	-0.58	372.1569,355.1532,323.1273,299.0911	Gomisin M1 isomer		WWZ	√		√	√
63	9.17	$C_{50}H_{84}O_{19}$	1033.5589	1033.5596	1.73	945.5324,783.4883,637.4318,475.3790	6"-Acetylginsenoside Rd isomer	PPT	HS	√	√		
64	9.32	$C_{44}H_{74}O_{15}$	887.501	887.5013	1.58	741.0736,637.4278,475.3785	6'-Acetyl-ginsenoside Rg 1 isomer	PPT	HS	√	√		
65	9.47	$C_{42}H_{72}O_{13}$	829.4955	829.4955	1.31	637.4301, 475.3808,403.3241	Ginsenoside Rg2 isomer	PPT	HS			√	
66#	9.49	$C_{23}H_{28}O_7$	417.1908	417.1906	-0.55	399.1813,375.1433,360.1196,345.1324,330.1088	Schisandrol B isomer		WWZ	√			
67	9.5	$C_{60}H_{102}O_{28}$	1269.6485	1269.6475	0.06	1107.5914,945.5394,783.4931,621.4406,459.3873	Ginsenoside Ra0	PPD	HS	√			
68	9.55	$C_{58}H_{79}O_{19}$	1077.5053	1077.512	5.79	899.4653,753.4061,607.3486	/		MD				
69	9.72	$C_{59}H_{100}O_{27}$	1285.6434	1285.6425	0.12	1239.6408,1107.5954,945.5436,783.4888,621.4371,459.3840	Notoginsenoside Fa	PPD	HS	√	√		√
70	9.85	$C_{45}H_{72}O_{19}$	927.4595	927.46	1.74	735.3948,555.3317	Ac-Ophiopojaponin C		MD	√	√	√	√
71*	9.93	$C_{42}H_{72}O_{14}$	845.4904	845.4899	0.73	799.4842,637.4320,475.3789	Ginsenoside R*f**	PPT	HS	√	√	√	√
72	10.04	$C_{60}H_{102}O_{28}$	1269.6485	1269.6472	-0.13	1107.5893,945.5354,783.4923,621.4366,459.3837	Notoginsenoside R4	PPD	HS	√	√		
73*	10.05	$C_{42}H_{72}O_{14}$	845.4904	845.4903	1.17	799.4875,637.4323,491.3754,475.3786	Pseudoginsenoside F11*	OT	HS	√	√	√	√
74	10.18	$C_{42}H_{72}O_{14}$	845.4904	845.4901	0.94	799.4841,637.4312,475.3789	Notoginsenoside K	PPT	HS	√		√	
75*	10.35	$C_{36}H_{62}O_{10}$	699.4325	699.4323	1.21	491.3745,441.7187	Pseudoginsenoside RT5*	OT	HS	√	√	√	
76	10.37	$C_{54}H_{90}O_{23}$	1151.5855	1151.5852	0.71	1105.5749,943.5244,781.4751,619.4205,457.3681	5, 6-Didehydroginsenoside Rb1		HS	√	√		
77	10.42	$C_{59}H_{100}O_{27}$	1285.6434	1285.6423	0.02	1239.6367,1077.5859,945.5455,783.4874,621.4368,459.3824	Ginsenoside Ra3 isomer	PPD	HS	√			

78	10.48	$C_{45}H_{72}O_{17}$	883.4697	883.4666	-2.23	837.4428,705.4069,637.4339,475.3785	Rha-Floribundasaponin B		MD	√	√		√
79*	10.49	$C_{41}H_{70}O_{13}$	815.4798	815.4796	0.98	769.4772,637.4315,475.3787	Notoginsenoside R2*	PPT	HS	√	√	√	√
80*	10.56	$C_{58}H_{98}O_{26}$	1255.6328	1255.6316	-0.12	1209.5982,1077.5832,945.5438,945.5438,621.4367,459.3844	Ginsenoside Ra2*	PPD	HS	√	√		√
81*	10.7	$C_{59}H_{100}O_{28}$	1285.6434	1285.642	-0.26	1239.6394,1107.5960,945.5446,783.4896,621.4367,459.3832	Ginsenoside Ra3*	PPD	HS	√	√		√
82#	10.74	$C_{23}H_{28}O_{8}$	433.1857	433.1852	-1.19	415.1742,400.1504,385.1222,347.1122	Gomisin P		WWZ	√		√	√
83*	10.79	$C_{54}H_{92}O_{23}$	1107.5957	1107.5949	0.26	945.5419,783.4896,621.4368,459.3840	Ginsenoside Rb1*	PPD	HS	√	√		√
84	10.8	$C_{37}H_{44}O_{7}$	599.3003	599.2993	-0.95	459.3819	/	PPD	HS				
85	11.02	$C_{42}H_{72}O_{15}$	815.4798	815.4799	1.44	637.4315,475.3789,391.2861	Ginsenoside Re5	PPT	HS	√		√	√
86	11.02	$C_{48}H_{76}O_{19}$	955.4908	955.4899	0.17	793.4375,749.4476,455.3528	Ginsenoside Ro isomer	OA	HS	√			
87*	11.05	$C_{42}H_{72}O_{13}$	829.4955	829.4954	1.23	529.3827,475.3787,459.3468	Ginsenoside Rg2(S)*	PPT	HS	√	√	√	√
88	11.07	$C_{57}H_{94}O_{26}$	1193.5961	1193.5955	0.42	1107.5982,945.5411,783.4893,621.4368,459.3842	Malonyl-ginsenoside Rb	PPD	HS	√	√		
89*	11.25	$C_{58}H_{98}O_{26}$	1255.6328	1255.6311	-0.51	1209.6148,1077.5828,945.5405,783.4888,621.4361,459.3842	Ginsenoside Ra1*	PPD	HS	√	√		√
90	11.28	$C_{42}H_{72}O_{13}$	829.4955	829.4952	1.02	783.4829,637.4307,475.3789,459.3438,391.2861	Ginsenoside Rg2(R)	PPT	HS	√	√	√	√
91*	11.28	$C_{36}H_{62}O_{9}$	683.4376	683.4371	0.85	637.4318,475.3790,459.3501,391.2857	Ginsenoside h1(S) *	PPT	HS	√	√		√
92	11.31	$C_{42}H_{66}O_{14}$	793.4368	793.4375	0.61	631.3848,569.3843,455.3520	isomer of chicusetsusaponin IV	OA	HS				
93	11.33	$C_{72}H_{184}O_{11}$	1123.5929	1123.5903	-2.66	1077.5853,945.5451,783.4915,621.4368,459.3841	PPT-20-glc(2→1)glc-6-glc-glc	PPD	HS				
94*	11.42	$C_{48}H_{76}O_{19}$	955.4908	955.4902	0.49	793.4373,731.4374,613.3737,569.3845,455.3535	Ginsenoside Ro*	OA	HS	√	√	√	√
95#	11.49	$C_{23}H_{28}O_{6}$	401.1959	401.1957	-0.46	386.1718,370.1765,337.1430,323.1271	Gomisin N isomer		WWZ	√			
96#	11.49	$C_{23}H_{30}O_{7}$	419.2064	419.2062	-0.67	386.1690,370.1765,355.1530,337.1430,323.1271	Gomisin H isomer		WWZ	√		√	√
97	11.59	$C_{58}H_{98}O_{26}$	1209.6274	1209.6268	0.47	1077.5837,945.5383,783.4887,621.4370,459.3840	Notoginsenoside Fc or Fp2	PPD	HS	√			
98	11.63	$C_{56}H_{92}O_{25}$	1163.5855	1163.5855	0.91	1077.5855,945.5424,783.4897,621.4357,459.3845	ManoylGinsenoside Rc	PPD	HS	√	√		
99	11.66	$C_{56}H_{94}O_{24}$	1195.6117	1195.6119	1.11	1107.5943,945.5420,783.4893,621.4373,459.3845	6''-*O*-acetylginsenoside Rb1 isomer	PPD	HS	√			

100	11.7	$C_{42}H_{72}O_{14}$	845.4904	845.4908	1.74	799.4858,637.4323,537.3431,475.3789,375.2903	Notoginsenoside K isomer	PPT	HS			√	
101#	11.7	$C_{23}H_{28}O_{6}$	401.1959	401.1957	-0.54	386.1719,370.1763,355.1529,338.1496,332.1245	Gomisin N isomer		WWZ	√			
102#	11.7	$C_{23}H_{30}O_{7}$	441.1884	441.3724	-0.67	423.3617,367.2990			WWZ	√			√
103	11.73	$C_{58}H_{98}O_{26}$	1209.6274	1209.6265	0.17	1143.6195,1077.5867,945.5435,783.4909,621.4366,459.3834	PPD-20-glc-xyl-xyl-3-glc-glc	PPD	HS	√			
104*	11.75	$C_{36}H_{62}O_{9}$	683.4376	683.4374	1.39	637.4304,553.3380,475.3790,391.2858`	Ginsenoside f1*	PPT	HS	√	√		√
105*	11.87	$C_{53}H_{90}O_{22}$	1123.5906	1123.5902	0.65	1077.5812,945.5382,783.4902,621.4368,459.3846	Ginsenoside Rb2*	PPD	HS	√	√		√
106	11.94	$C_{47}H_{74}O_{18}$	925.4802	925.4802	1.1	763.4268,701.4267,613.3739,569.3843,523.3786,455.3524	Saponin Rb-2 isomer	OA	HS	√	√	√	√
107*	12.05	$C_{53}H_{90}O_{22}$	1123.5906	1123.5905	0.86	1077.6015,945.5358,783.4901,621.4363,459.3851	Ginsenoside Rb3*	PPD	HS	√	√		√
108	12.15	$C_{44}H_{74}O_{15}$	887.501	887.5015	1.86	391.2856,475.3787,619.4231,637.4330,799.4798	6'-Acetyl-ginsenoside Rg 1 isomer	PPT	HS	√	√		
109	12.17	$C_{56}H_{92}O_{25}$	1163.5855	1163.5858	1.23	459.3841,621.4358,783.4892,945.5429,1077.5935	ManoylGinsenoside Rb2	PPD	HS	√	√		
110	12.17	$C_{58}H_{98}O_{26}$	1255.6328	1255.6325	0.57	1209.6329,1077.5835,945.5437,783.4893,621.4370,459.3845	PPD-20-glc-xyl-xyl-3-glc-glc	PPD	HS	√			
111#	12.23	$C_{24}H_{32}O_{8}$	449.217	449.2169	-0.23	431.2083,416.1815,400.1875,382.1762,373.1644,358.1393,342.1457,327.1221	7,8-Dihydroxy-schisandrin		WWZ	√		√	√
112	12.26	$C_{47}H_{74}O_{18}$	925.4802	925.481	1.97	763.4267,701.4265,613.3741,455.3528	Saponin Rb-2	OA	HS	√	√	√	√
113	12.35	$C_{55}H_{92}O_{23}$	1165.6011	1165.6014	1.2	1077.5819,945.5434,783.4890,621.4380,459.3837	Ginsenoside Rs1 isomer	PPD	HS	√			
114	12.5	$C_{47}H_{74}O_{18}$	925.4802	925.4806	1.57	793.4386,613.3750,569.3845,455.3535	Saponin Rb-2 isomer	OA	HS	√	√	√	√
115#	12.52	$C_{23}H_{28}O_{6}$	401.1959	401.1957	-0.39	386.1720,370.1766,355.1534,332.1244	Gomisin N		WWZ	√			
116#	12.52	$C_{23}H_{30}O_{7}$	419.2064	419.2062	-0.6	401.1946,386.1728,370.1766,355.1530,338.1500,332.1247,318.1088	Gomisin H isomer		WWZ	√		√	√
117	12.57	$C_{56}H_{94}O_{24}$	1195.6117	1195.6116	0.8	1107.5936,945.5482,783.4906,621.4360,459.3845	6"-*O*-acetylginsenoside Rb1	PPD	HS	√	√		
118	12.69	$C_{53}H_{90}O_{23}$	1093.58	1093.5803	1.29	915.5251,753.4817,621.4367,459.3836	Floralginsenoside P	PPD	HS	√	√		
119	12.76	$C_{56}H_{94}O_{24}$	1195.6117	1195.6115	0.7	1107.6005,945.5427,783.4888,621.4366,459.3838	6"-*O*-acetylginsenoside Rb1 isomer	PPD	HS	√			

120	12.78	$C_{50}H_{77}O_{20}$	997.5014	997.5014	1.13	835.4478,773.4484,713.4279,569.3844,523.3788,455.3525	Xyl-Ophiopogonin D isomer		MD	√	√		√
121#	12.87	$C_{23}H_{28}O_6$	401.1959	401.1959	0.06	386.1714,370.1765,337.1430,323.1272	Gomisin N isomer		WWZ	√			
122#	12.87	$C_{23}H_{30}O_7$	419.2064	419.2064	-0.1	401.1973,386.1712,370.1769,355.1523,337.1430,323.1274	Gomisin H		WWZ	√		√	√
123	12.89	$C_{55}H_{92}O_{23}$	1165.6011	1165.6016	1.3	1077.5871,945.5452,783.4876,621.4375,459.3831	Ginsenoside Rs1 isomer	PPD	HS	√			
124*	12.92	$C_{36}H_{62}O_9$	683.4376	683.4376	1.66	475.3783	20(*R*)-ginsenoside Rh1*	PPT	HS	√	√		
125	13.05	$C_{56}H_{94}O_{24}$	1195.6117	1195.6118	1.01	1107.5898,945.5561,783.4877,621.4369,459.3850	6"-*O*-acetylginsenoside Rb1 isomer	PPD	HS	√			
126#	13.16	$C_{23}H_{28}O_6$	401.1959	401.1959	0.14	386.1718,370.1766,355.1532,332.1247	Gomisin N isomer		WWZ	√			
127*	13.28	$C_{48}H_{82}O_{18}$	991.5483	991.5482	1.01	945.5416,783.4915,621.4364,459.3845	Ginsenoside Rd*	PPD	HS	√	√		
128	13.32	$C_{42}H_{66}O_{14}$	793.438	793.4375	0.78	631.3848,569.3843,455.3520	Chikusetsu saponin IVa	OA	HS	√	√		
129	13.32	$C_{55}H_{92}O_{23}$	1165.6011	1165.6005	0.36	1077.5798,945.5439,783.4890,621.4359,459.3848	PPD-20-glc-xyl-3-glc-glc-Ac	PPD	HS	√			
130#	13.51	$C_{23}H_{28}O_6$	401.1959	401.1958	-0.24	386.1733,370.1767,355.1532,332.1246	Gomisin N isomer		WWZ	√			
131	13.55	$C_{44}H_{70}O_{18}$	931.4544	931.4548	1.56	885.4453,753.4072,607.3499,445.2955	Ophiopogonin C*		MD	√	√	√	√
132	13.58	$C_{55}H_{92}O_{23}$	1165.6011	1165.6007	0.57	1077.5828,945.5412,783.4903,621.4369,459.3833	Ginsenoside Rs1 isomer	PPD	HS	√			
133	13.61	$C_{51}H_{84}O_{21}$	1031.5432	1031.5435	1.28	945.5457,783.4887,621.4364,459.3846	Malonyl-ginsenoside Rd	PPD	HS	√	√		
134	13.79	$C_{51}H_{84}O_{21}$	1031.5432	1031.5441	1.88	945.5298,783.4863,621.4368,459.3836	Malonyl-ginsenoside Rd isomer	PPD	HS	√	√		
135	13.88	$C_{56}H_{94}O_{24}$	1195.6117	1195.6118	1.01	1107.5980,945.5375,783.4893,621.4368,459.3856	6"-*O*-acetylginsenoside Rb1 isomer	PPD	HS	√			
136	13.92	$C_{55}H_{92}O_{23}$	1165.6011	1165.6017	1.4	1077.5844,945.5416,783.4898,621.4365,459.3846	Ginsenoside Rs1	PPD	HS	√			
137	14.01	$C_{40}H_{64}O_{16}$	799.411	799.4121	1.24	753.4111,607.3522,445.2962	Ophiogenin 3-*O*-*α*-L-rhamnopyranosyl-(1→2)-*β*-D-glucopyranoside*	Type II	MD	√	√	√	√
138	14.1	$C_{55}H_{92}O_{23}$	1165.6011	1165.6017	1.4	1077.5838,945.5384,783.4896,621.4373,459.3836	Ginsenoside Rs1 isomer	PPD	HS	√			
139	14.17	$C_{53}H_{90}O_{23}$	1093.58	1093.5806	1.51	621.4376,459.3807	Floralginsenoside P isomer	PPD	HS	√	√		
140	14.46	$C_{50}H_{77}O_{20}$	997.5014	997.5018	1.56	835.4486,731.4381,569.3843,523.3789,455.3523	Xyl-Ophiopogonin D		MD	√	√		√

141#	14.46	$C_{23}H_{30}O_7$	419.2064	419.2064	-0.02	401.1937,386.1727,370.1767,355.1528,338.1507,323.1274	Gomisin T		WWZ	√		√	√
142	14.47	$C_{55}H_{92}O_{23}$	1165.6011	1165.6018	1.52	1077.5793,945.5368,783.4930,621.4371,459.3822	Ginsenoside Rs1 isomer	PPD	HS	√			
143	14.58	$C_{50}H_{84}O_{19}$	1033.5589	1033.5592	1.37	945.5444,783.4874,621.4378,459.3835	6"-Acetylginsenoside Rd isomer	PPD	HS	√			√
144#	14.6	$C_{24}H_{32}O_7$	455.204	455.2039	-0.36	440.1836,427.1695,409.1621,318.9522	Gomisin S		WWZ	√			√
145	14.62	$C_{55}H_{92}O_{23}$	1165.6011	1165.6016	1.3	1077.5832,945.5446,783.4917,621.4353,459.3837	Ginsenoside Rs1 isomer	PPD	HS	√			
146	14.86	$C_{30}H_{36}O_{13}$	603.2072	603.2084	1.22	499.1600,471.1683,455.1733	/	OA	HS				
147	14.87	$C_{53}H_{90}O_{23}$	1093.58	1093.5802	1.18	621.4411	Floralginsenoside P isomer		HS	√	√		
148	14.98	$C_{47}H_{80}O_{17}$	961.5378	961.538	1.42	915.52142,753.4780,621.4366,459.3843	Ginsenoside Rd 2 isomer	PPD	HS	√			
149	15.28	$C_{55}H_{92}O_{23}$	1165.6011	1165.6017	1.4	1077.5822,945.5465,783.4896,621.4389,459.3863	Ginsenoside Rs1 isomer	PPD	HS	√			
150#	15.34	$C_{23}H_{28}O_6$	401.1959	401.1958	-0.24	386.1718,370.1764,355.1531,332.1247	Gomisin N isomer		WWZ	√			
151	15.58	$C_{47}H_{80}O_{17}$	961.5378	961.538	1.36	915.5283,783.4912,621.4365,459.3841	Ginsenoside Rd 2	PPD	HS	√			
152	15.69	$C_{50}H_{84}O_{19}$	1033.5589	1033.5593	1.49	945.5476,783.4923,621.4375,459.3841	6"-Acetylginsenoside Rd	PPD	HS	√	√		√
153	15.74	$C_{47}H_{80}O_{17}$	961.5378	961.5382	1.62	915.5360,783.4849,621.4364,459.3845	Ginsenoside Rd 2 isomer	PPD	HS	√			
154	15.83	$C_{38}H_{60}O_{14}$	785.3965	785.3973	2.46	739.4203,445.2950	Ophiogenin 3-O-xyl(1→4) β-D-glc		MD	√			√
155#	16.12	$C_{22}H_{26}O_6$	387.1802	387.18	-0.58	372.1565,355.1532,323.1274,299.0911	Gomisin M1 isomer		WWZ	√		√	√
156	16.17	$C_{47}H_{80}O_{17}$	961.5378	961.5382	1.62	621.4365,459.3853	Ginsenoside Rd 2 isomer	PPD	HS	√			
157	16.24	$C_{41}H_{68}O_{12}$	797.4693	797.4695	1.63	619.4234,527.3751, 457.3690	Hebevinoside VI		HS	√	√	√	√
158	16.24	$C_{50}H_{84}O_{19}$	1033.5589	1033.5592	1.37	945.5405,783.4836,621.4360,459.3842	6"-Acetylginsenoside Rd isomer	PPD	HS	√			
159	16.26	$C_{44}H_{70}O_{17}$	915.4595	915.46	1.76	737.4132,525.3126	(25R)-Ruscogenin 3-yl α-L-Rha-(1→2)-[β-D-Xyl-(1→4)]-β-D-Glc		MD	√	√	√	
160	16.33	$C_{50}H_{84}O_{19}$	1033.5589	1033.5593	1.49	945.5402,783.4875,621.4378,459.3836	6"-Acetylginsenoside Rd isomer	PPD	HS	√			
161	16.48	$C_{44}H_{70}O_{17}$	915.4595	915.4601	1.83	815.4480,737.4130,591.3585,463.0913	Pennogenin 3-*O*-*α*-Lrhamnopyranosyl-(1→2)-*β*-D		MD	√	√	√	

							-xylopyranosyl-(1→4)-β-D-glucopyra noside isomer						
162	16.62	$C_{42}H_{70}O_{12}$	811.4849	811.4851	1.57	619.4241,553.3859,499.3786,499.3786,457.3728	Ginsenoside Rg6		HS	√	√	√	
163	16.62	$C_{44}H_{70}O_{17}$	915.4595	915.4597	1.44	737.4107,525.3241	Pennogenin 3-*O*-*α*-Lrhamnopyranosyl-(1→2)-*β*-D -xylopyranosyl-(1→4)-*β*-D-glucopyra noside		MD	√	√	√	
164	16.71	$C_{42}H_{70}O_{12}$	811.4849	811.4851	1.5	765.4687,619.4157,499.3713,457.3657	Ginsenoside Rg4		HS	√	√		
165#*	16.79	$C_{24}H_{32}O_{7}$	455.204	455.2036	-1.04	440.1820, 425.1582, 409.1593	Schisandrin*		WWZ	√	√	√	√
166	16.8	$C_{50}H_{84}O_{19}$	1033.5589	1033.5592	1.37	945.5449,783.4909,621.4370,459.3845	6''-Acetylginsenoside Rd isomer	PPD	HS	√			√
167	16.81	$C_{41}H_{68}O_{12}$	797.4693	797.4694	1.55	619.4212,457.3656	Hebevinoside VI isomer		HS	√	√	√	√
168	17.12	$C_{50}H_{84}O_{19}$	1033.5589	1033.5596	1.73	945.5457,783.4897,621.4365,459.3848	6''-Acetylginsenoside Rd isomer	PPD	HS	√			
169	17.19	$C_{39}H_{62}O_{13}$	783.4172	783.4172	1.4	737.4134	Polyphyllin Ⅵ isomer		MD	√	√		√
170	17.22	$C_{42}H_{70}O_{12}$	811.4849	811.4849	1.34	765.4768,619.4248,457.3705,391.3643	Ginsenoside Rg5		HS	√		√	
171#	17.32	$C_{23}H_{28}O_{7}$	417.1908	417.1905	-0.77	401.1928,385.1987,370.1728,353.1376,342.1459,329.1374,314.1139	Schisandrol B isomer		WWZ	√			√
172	17.33	$C_{39}H_{62}O_{13}$	783.4172	783.4172	1.4	737.4136	Polyphyllin Ⅵ		MD	√	√		
173#*	18.08	$C_{28}H_{34}O_{10}$	553.2044	553.2042	-0.47	535.1902,507.1987,466.3147,449.1545, 391.1145, 376.1277	Gomisin D*		WWZ	√	√	√	√
174*	18.17	$C_{42}H_{72}O_{13}$	829.4955	829.4952	1.02	793.4379,613.3748,569.3841,523.3776,455.3541	Ginsenoside f2*	OA	HS	√	√		
175#	18.3	$C_{22}H_{28}O_{6}$	389.1959	389.1957	-0.32	374.1716,357.1693,325.1431,287.0910,227.0700	Gomisin J		WWZ	√	√	√	√
176#*	18.62	$C_{23}H_{28}O_{7}$	439.1727	439.1724	-0.83	424.1500, 405.3629,345.4505,303.1373,298.4059	Schisandrol B*		WWZ	√	√	√	√
177#	18.75	$C_{24}H_{32}O_{7}$	455.204	455.2037	-0.78	440.1820, 425.1572,409.1629,402.4775,311.0787	Isoschisandrin		WWZ	√			
178	18.78	$C_{41}H_{64}O_{13}$	763.4274	763.4276	1.61	701.4219,613.3746,569.3838,455.3525	Ophiopogonin A isomer		MD	√			√
179*	18.95	$C_{42}H_{72}O_{13}$	829.4955	829.4955	1.31	783.4874,621.4370,459.3844	Ginsenoside Rg3*	PPD	HS	√	√	√	√

180	19.06	$C_{44}H_{74}O_{14}$	871.5061	871.5056	0.75	783.4902,621.4385,459.3838	Ginsenoside Rs3 isomer	PPD	HS	√			
181	19.13	$C_{42}H_{72}O_{13}$	829.4955	829.4955	1.31	783.4882,621.4375,459.3838	Ginsenoside Rg3 isomer	PPD	HS	√	√	√	√
182	19.21	$C_{44}H_{74}O_{14}$	871.5061	871.5057	0.82	783.4903,621.4365,459.3842	Ginsenoside Rs3 isomer	PPD	HS	√			
183#	19.27	$C_{28}H_{34}O_{9}$	515.2276	515.2277	0.31	486.2717,446.1551,422.5422,400.1508,385.1646,370.1410,355.1532,316.0937	Schizantherin C isomer		WWZ	√			
184#	19.3	$C_{24}H_{32}O_{7}$	455.204	455.2038	-0.49	440.1829,425.1565,409.1633,340.0173	Isoschisandrin		WWZ	√			
185	19.37	$C_{41}H_{64}O_{13}$	763.4274	763.4277	1.85	631.3860,569.3843,455.3519	Ophiopogonin A		MD	√	√		√
186	19.4	$C_{42}H_{72}O_{13}$	829.4955	829.4963	2.27	783.4871, 621.4356,459.3843	Ginsenoside Rg3 isomer	PPD	HS			√	
187#	19.45	$C_{28}H_{34}O_{9}$	515.2276	515.2278	0.43	469.2222,438.2023,400.1520,385.1639,370.1403,355.1529,316.0936	Schizantherin C		WWZ	√	√	√	√
188#	19.52	$C_{23}H_{28}O_{6}$	401.1959	401.1957	-0.54	386.1713,370.1766,355.1531,338.1499,332.1246	G-schisandrin isomer		WWZ	√	√	√	√
189#	19.52	$C_{28}H_{36}O_{8}$	523.2302	523.23	-0.38	508.2047,493.1821,477.1857,409.1618,386.1722	Angeloylgomisin H		WWZ	√	√	√	√
190*	19.56	$C_{44}H_{70}O_{16}$	899.4643	899.4648	1.5	721.4169,575.3591,411.2894	Ophiopogonin D*		MD	√	√	√	√
191	19.65	$C_{44}H_{70}O_{16}$	899.4643	899.4651	1.78	721.4123,575.35754	Ophiopogonin D'		MD	√			
192	19.67	$C_{42}H_{72}O_{12}$	813.5006	813.5011	1.99	707.2197,605.4448	Ginsenoside Rk1		HS	√			√
193	19.67	$C_{42}H_{72}O_{13}$	829.4955	829.4965	2.49	783.4819,621.4345,459.3827	Ginsenoside Rg3 isomer	PPD	HS			√	
194#	19.74	$C_{23}H_{28}O_{6}$	401.1959	401.1957	-0.31	386.1716,370.1757,355.1526,332.1241	G-schisandrin isomer		WWZ	√	√	√	√
195#	19.76	$C_{23}H_{28}O_{7}$	439.1727	439.1726	-0.4	424.1475,409.1230,393.1284	Schisandrol B isomer		WWZ	√		√	√
196	19.77	$C_{44}H_{74}O_{14}$	871.5061	871.5064	1.59	783.4890,621.4355,459.3837,375.2909	Ginsenoside Rs3 isomer	PPD	HS	√			√
197	19.85	$C_{44}H_{70}O_{16}$	899.4643	899.4652	1.84	721.4178	OJV-Ⅵ		MD	√		√	√
198	19.87	$C_{36}H_{56}O_{9}$	631.384	631.3851	1.13	555.3682,509.3638,455.3526	/	OA	HS				
199	19.94	$C_{44}H_{74}O_{14}$	871.5061	871.5064	1.66	783.4900,621.4381,459.3843	Ginsenoside Rs3 isomer	PPD	HS	√			
200#	20	$C_{23}H_{28}O_{6}$	401.1959	401.1956	-0.69	386.1724,370.1764,355.1531,332.1247	G-schisandrin		WWZ	√	√	√	√

201#	20	$C_{28}H_{36}O_8$	523.2302	523.2299	-0.61	508.2080,493.1812,477.1877,409.1623,386.1711	Tigloylgomisin H		WWZ	√	√	√	√
202#	20.03	$C_{29}H_{38}O_9$	553.2408	553.2404	-0.69	453.1881,431.2053,400.1872,387.1804,372.1563,356.1612	TigloylgomisinQ/Angeloylgomisin Q		WWZ	√	√	√	
203	20.07	$C_{44}H_{74}O_{14}$	871.5061	871.5061	1.31	783.4926,621.4371,459.3836	Ginsenoside Rs3	PPD	HS	√	√	√	√
204#	20.17	$C_{23}H_{28}O_7$	439.1727	439.1726	-0.19	424.1475, 393.1290,299.2580,278.4048,268.2644	Schisandrol B isomer		WWZ	√		√	√
205	20.21	$C_{44}H_{74}O_{14}$	871.5061	871.5064	1.59	783.4899,621.4369,459.3843	Ginsenoside Rs3 isomer	PPD	HS	√	√	√	√
206	20.25	$C_{42}H_{72}O_{12}$	813.5006	813.5007	1.53	767.4901,605.4413,	Ginsenoside Rg5		HS	√			
207	20.34	$C_{33}H_{55}O_{14}$	723.3809	723.3808	1.44	397.1353	Gingerglycolipid B		HS	√	√	√	√
208#	20.39	$C_{29}H_{38}O_9$	553.2408	553.2404	-0.69	453.1879,431.2070,416.1845,400.1878,382.1771,372.1560,356.1614	Benzoylgomisin Q		WWZ	√	√	√	
209	20.48	$C_{44}H_{74}O_{14}$	871.5061	871.506	1.24	783.4906,621.4328,459.3827	Ginsenoside Rs3 isomer	PPD	HS	√			
210	20.49	$C_{37}H_{60}O_{10}$	663.4102	663.4113	0.49	455.3528	/	OA	HS				
211#	20.6	$C_{28}H_{34}O_9$	537.2095	537.2093	-0.4	437.1566,415.1740,400.1508,384.1564,371.1489,356.1249,340.1300	Schizantherin B		WWZ	√		√	√
212	20.62	$C_{44}H_{74}O_{14}$	871.5061	871.5064	1.59	783.4895,621.4371,459.3844	Ginsenoside Rs3 isomer	PPD	HS	√			
213	20.68	$C_{42}H_{70}O_{12}$	811.4849	811.4848	1.19	765.4708,603.4262,537.3917	Ginsenoside Rk1		HS	√	√	√	√
214	20.69	$C_{44}H_{72}O_{13}$	853.4955	853.4956	1.42	765.4805,603.4245,476.2064	Ginsenoside Rs5 isomer		HS	√			
215	20.85	$C_{42}H_{70}O_{12}$	811.4849	811.4847	1.12	765.4806,603.4229,537.3947,511.3799	Ginsenoside Rz1		HS	√	√	√	√
216	20.87	$C_{44}H_{72}O_{13}$	853.4955	853.4955	1.27	765.4760,603.4236	Ginsenoside Rs5 isomer		HS	√			
217#	21.07	$C_{28}H_{34}O_9$	537.2095	537.2094	-0.17	437.1570,415.1750, 400.1507, 385.1647,371.1484,356.1246,340.1301	Schizantherin C		WWZ	√	√	√	√
218	21.18	$C_{44}H_{72}O_{13}$	853.4955	853.4957	1.56	765.4744	Ginsenoside Rs5 isomer		HS	√			
219*	21.19	$C_{36}H_{62}O_8$	667.4427	667.4429	1.94	497.3412,459.3854,375.2913	Ginsenoside CK*	PPD	HS	√	√		√

220*	21.39	$C_{36}H_{62}O_8$	667.4427	667.4429	2.03	505.1097, 459.3853, 307.2635	Ginsenoside Rh2*	PPD	HS	√	√	√	√
221	21.54	$C_{36}H_{62}O_8$	667.4427	667.443	2.12	569.9765,459.3823	PPD-glc	PPD	HS	√	√	√	√
222	21.61	$C_{44}H_{72}O_{13}$	853.4955	853.496	1.84	807.4893,765.4774,603.4276,537.3957	Ginsenoside Rs5 isomer		HS	√			
223	21.7	$C_{44}H_{72}O_{13}$	853.4955	853.4957	1.56	765.4772,603.4276,483.3876	Ginsenoside Rs5		HS	√	√	√	
224	21.86	$C_{44}H_{72}O_{13}$	853.4955	853.4955	1.27	765.4801,603.4262,537.3972	Ginsenoside Rs4		HS	√	√	√	
225	21.95	$C_{44}H_{72}O_{13}$	853.4955	853.4956	1.42	765.4789,603.4310,537.3995	Ginsenoside Rs5 isomer		HS	√			
226	22.1	$C_{44}H_{72}O_{13}$	853.4955	853.4959	1.77	765.4829,603.4287	Ginsenoside Rs5 isomer		HS	√			
227#*	22.32	$C_{24}H_{32}O_6$	417.2272	417.2273	0.16	402.2034,386.2086,371.1841,316.1302,301.1066	Schisanhenol *		WWZ	√	√	√	√

Note: * Compared by reference standard; # detected in positive ion mode; HS, Hongshen; WWZ, Wuweizi; MD, Maidong; a, YQFMI; b, SMJN; c, SMY; d, YQJN; PPD, 20(S)-Protopanaxadiol; PPT, 20(S)-protopanaxatriol; OT, octillol; OA, oleanolic acid; /, not identified.

Table S3. Information of 13 important variables showed higher inter-group variance.

Variable number	VIP value	Compound name	Compound type	Source
V113	1.25	ginsenoside Rf	ginsenoside	HS
V214	1.21	ginsenoside Rg2	ginsenoside	HS
V337	1.35	ophiopogonin D	steroidal saponin	MD
V338	1.34	ginsenoside Re	ginsenoside	HS
V441	1.42	ginsenoside Rg1	ginsenoside	HS
V472	1.21	ginsenoside F11	ginsenoside	HS
V540	1.40	ginsenoside Rd	ginsenoside	HS
V218	1.21	schisandrol	lignan	WWZ
V235	1.35	schisandrin C	lignan	WWZ
V459	1.25	schisandrin	lignan	WWZ
V451	1.14	gomisin D	lignan	WWZ
V696	1.26	gomisin H	lignan	WWZ
V762	1.26	schisandrol B	lignan	WWZ

HS (Hongshen, Ginseng Radix et Rhizoma Rubra), MD (Maidong, Ophiopogonis Radix), and WWZ (Wuweizi, Schisandrae Chinensis Fructus)

Table S4. Linear regression data of the 25 analytes.

No.	Linearity	r^2	Initial concentration (ng/mL)	LOQ (ng/mL)
R1	Y = -0.0196068+0.00951539*X+2.3748e-7*X^2	0.9987	4950.00	2.42
R2	Y = -0.00596513+0.000212457*X-1.02966e-8*X^2	0.9987	4982.00	38.92
R3	Y = -0.00090726+0.000473076*X-3.13588e-8*X^2	0.9986	5005.00	39.10
R4	Y = -0.0032546+0.000810495*X+3.54383e-8*X^2	0.9992	5005.00	39.10
R5	Y = -0.000221198+0.00124392*X+3.14448e-7*X^2	0.9991	4998.00	9.67
R6	Y = -0.00719303+0.000181903*X+1.11724e-8*X^2	0.9983	4995.00	156.09
R7	Y = 0.013123+0.000203872*X+8.92349e-8*X^2	0.9980	4992.00	156.00
R8	Y = 0.0154618+0.0025645*X+7.13924e-7*X^2	0.9972	4981.50	9.73
R9	Y = 0.0054395+0.00382838*X+1.54331e-6*X^2	0.9974	4950.00	9.67
R10	Y = 0.00462219+0.000116629*X+4.21704e-8*X^2	0.9974	5005.00	156.41
R11	Y = -0.0554654+0.000124342*X-9.61416e-9*X^2	0.9941	4992.00	38.92
R12	Y = -0.641748+0.00591365*X+1.63922e-6*X^2	0.9964	5022.00	9.81
R13	Y = 0.00276205+0.00410326*X+1.39714e-6*X^2	0.9976	4995.50	0.61
R14	Y = 0.0406786+0.000757848*X-2.84252e-8*X^2	0.9973	5002.50	39.08
R15	Y = 0.0109261+0.00308457*X+1.62926e-6*X^2	0.9961	4998.00	9.76

R16	Y = -0.00265359+0.00256623*X+9.44422e-7*X^2	0.9963	4982.00	9.73
R17	Y = -0.00325856+0.00497086*X+1.20028e-6*X^2	0.9969	5000.00	9.77
R18	Y = 0.00934972+0.00113488*X+8.35027e-8*X^2	0.9978	4992.00	2.43
R19	Y = -0.0296772+0.0188471*X-1.03911e-7*X^2	0.9991	5000.00	2.44
R20	Y = -0.0130326+0.0211252*X+4.03617e-7*X^2	0.9990	4999.50	0.61
R21	Y = -0.0339612+0.0455311*X+2.71669e-6*X^2	0.9976	5002.50	0.15
R22	Y = -0.0485177+0.0094071*X-5.74567e-10*X^2	0.9925	5005.00	2.44
R23	Y = -0.0315455+0.173508*X+1.82023e-6*X^2	0.9981	4972.00	0.15
R24	Y = 0.00213445+0.00142898*X-5.24227e-8*X^2	0.9952	4998.00	9.76
R25	Y = 0.0093364+0.0724057*X-1.41067e-6*X^2	0.9993	4998.00	0.15

Note: In the regression equation, Y represents the peak area ratio of analyte over the internal standard ($A_{analyte}/A_{istd}$), X the analyte concentration (ng/mL), and *r* the correlation coefficient.

Table S5. Repeatability, precision and stability variations of 25 analytes.

No.	Precision (RSD)		Stability	Repeatability
	Intra-day	Inter-day	RSD	RSD
R1	0.37%	0.53%	1.76%	1.21%
R2	0.39%	0.67%	2.85%	2.15%
R3	1.12%	1.99%	1.60%	1.34%
R4	0.50%	0.31%	4.05%	1.13%
R5	1.13%	1.27%	0.59%	2.32%
R6	1.36%	1.55%	3.33%	3.78%
R7	0.68%	0.19%	0.33%	0.48%
R8	0.88%	0.99%	0.82%	1.78%
R9	1.08%	2.14%	0.81%	4.21%
R10	0.84%	0.47%	1.13%	3.79%
R11	2.80%	1.65%	1.48%	3.66%
R12	1.67%	2.26%	2.73%	4.70%
R13	1.93%	2.40%	1.77%	3.62%
R14	0.39%	0.10%	0.27%	3.90%
R15	0.44%	0.34%	0.41%	3.59%
R16	0.37%	0.78%	0.46%	3.22%
R17	0.58%	0.90%	2.70%	3.85%
R18	0.67%	0.44%	4.06%	2.37%
R19	0.46%	0.15%	3.38%	1.77%
R20	0.50%	0.59%	0.74%	1.35%
R21	0.43%	0.58%	2.49%	2.65%
R22	0.46%	0.25%	0.35%	1.27%
R23	0.28%	0.57%	0.40%	2.85%
R24	0.77%	0.83%	0.94%	2.83%
R25	0.59%	0.23%	0.63%	1.99%

Table S6. Recovery of the analytes (n = 6).

No.	Found (ng)	Spiked (ng)	Mean Recovery (%, n=6)	RSD (%)
R1	20.18	19.80	101.88	2.88
R2	52.44	51.94	100.95	8.05
R3	691.69	699.40	98.90	4.92
R4	981.18	980.10	100.11	1.76
R5	398.48	399.84	99.66	2.91
R6	35.64	35.10	101.52	4.86
R7	168.44	169.52	99.36	5.02
R8	192.56	191.97	100.30	5.80
R9	1996.76	1999.80	99.85	3.58
R10	139.81	139.70	100.08	6.54
R11	252.32	250.04	100.91	1.05
R12	211.70	210.12	100.75	1.35
R13	591.92	599.85	98.68	1.53
R14	764.57	760.15	100.58	3.56
R15	721.36	721.14	100.03	2.08
R16	116.66	116.56	100.08	2.91
R17	1.56	1.50	103.42	0.52
R18	372.17	372.48	99.95	0.70
R19	36.46	36.36	100.28	3.59
R20	34.61	35.00	98.89	1.45
R21	140.90	141.45	99.60	3.81
R22	7.20	7.00	102.80	2.55
R23	14.98	15.21	98.43	4.53
R24	139.07	138.72	100.25	0.88
R25	0.32	0.30	108.10	0.42

Table S7. The contents of 25 analytes in 30 batches of SMS-based patent drugs.

No.	SMJN average ±SD (mg/0.30g, n=6)	SMY average ±SD (mg/10 mL, n=6)	YQFMI average ±SD (mg/0.65g, n=12)	YQJN average ±SD (mg/0.37g, n=6)
R1	0.01 ±0.00	/	0.01 ±0.00	/
R2	0.02 ±0.00	/	0.03 ±0.00	0.01 ±0.01
R3	0.46 ±0.01	/	0.39 ±0.07	0.41 ±0.44
R4	0.39 ±0.01	/	0.52 ±0.08	0.24 ±0.23
R5	0.13 ±0.00	0.01 ±0.00	0.25 ±0.03	0.06 ±0.05
R6	0.04 ±0.00	0.05 ±0.03	0.01 ±0.01	0.03 ±0.04
R7	0.05 ±0.00	/	0.10 ±0.02	0.02 ±0.02
R8	0.06 ±0.00	/	0.10 ±0.02	0.02 ±0.03
R9	0.94 ±0.02	/	1.03 ±0.19	0.26 ±0.43
R10	0.08 ±0.00	/	0.08 ±0.02	0.08 ±0.03
R11	0.14 ±0.00	0.05 ±0.11	0.11 ±0.08	0.12 ±0.02
R12	0.13 ±0.01	/	0.12 ±0.02	0.03 ±0.06
R13	0.26 ±0.01	/	0.28 ±0.06	0.10 ±0.11
R14	0.23 ±0.01	0.20 ±0.04	0.17 ±0.12	0.08 ±0.11
R15	0.30 ±0.00	/	0.34 ±0.06	0.14 ±0.12
R16	0.06 ±0.00	/	0.05 ±0.01	0.05 ±0.05
R17	/	/	/	0.04 ±0.06
R18	0.18 ±0.01	/	0.24 ±0.05	0.15 ±0.13
R19	/	/	0.02 ±0.00	/
R20	/	/	0.02 ±0.00	/
R21	0.01 ±0.00	0.78 ±0.05	0.09 ±0.01	0.46 ±0.23
R22	/	0.04 ±0.00	/	0.03 ±0.02
R23	/	0.11 ±0.01	0.01 ±0.00	0.06 ±0.03
R24	0.04 ±0.00	0.06 ±0.02	0.01 ±0.03	0.08 ±0.05
R25	/	0.01 ±0.00	/	/

Total	3.52±0.06	1.30±0.22	4.01±0.65	2.49±1.20

Table S8. Detailed information for the 30 batches of different SMS-based patent drugs.

No.	Name	Lot number
1	YQFM Injection (YQFMI-1)	20180511
2	YQFM Injection (YQFMI-2)	20180506
3	YQFM Injection (YQFMI-3)	20170808
4	YQFM Injection (YQFMI-4)	20171006
5	YQFM Injection (YQFMI-5)	20170809
6	YQFM Injection (YQFMI-6)	20180203
7	YQFM Injection (YQFMI-7)	20180503
8	YQFM Injection (YQFMI-8)	20180404
9	YQFM Injection (YQFMI-9)	20170701
10	YQFM Injection (YQFMI-10)	20180513
11	YQFM Injection (YQFMI-11)	20170811
12	YQFM Injection (YQFMI-12)	20180104
13	YQFM Capsule (YQJN-1)	20050101
14	YQFM Capsule (YQJN-2)	20050102
15	YQFM Capsule (YQJN-3)	20040101
16	YQFM Capsule (YQJN-4)	20040102
17	YQFM Capsule (YQJN-5)	200502
18	YQFM Capsule (YQJN-6)	200402
19	Shengmai Capsule (SMJN-1)	200400601
20	Shengmai Capsule (SMJN-2)	2004004
21	Shengmai Capsule (SMJN-3)	200600201
22	Shengmai Capsule (SMJN-4)	200600602
23	Shengmai Capsule (SMJN-5)	2008003
24	Shengmai Capsule (SMJN-6)	200600202
25	Shengmaiyin Oral Solution (SMY-1)	20260376
26	Shengmaiyin Oral Solution (SMY-2)	2026051601

27	Shengmaiyin Oral Solution (SMY-3)	20260406
28	Shengmaiyin Oral Solution (SMY-4)	2026051101
29	Shengmaiyin Oral Solution (SMY-5)	2026051602
30	Shengmaiyin Oral Solution (SMY-6)	2026051102
